# NADPH oxidase 1 is highly expressed in human large and small bowel cancers

**DOI:** 10.1371/journal.pone.0233208

**Published:** 2020-05-19

**Authors:** Jiamo Lu, Guojian Jiang, Yongzhong Wu, Smitha Antony, Jennifer L. Meitzler, Agnes Juhasz, Han Liu, Krishnendu Roy, Hala Makhlouf, Rodrigo Chuaqui, Donna Butcher, Mariam M. Konaté, James H. Doroshow

**Affiliations:** 1 Center for Cancer Research, National Cancer Institute, Bethesda, Maryland, United States of America; 2 Division of Cancer Treatment and Diagnosis, National Cancer Institute, Bethesda, Maryland, United States of America; 3 Pathology/Histotechnology Laboratory, Leidos Biomedical Research, Inc., Frederick National Laboratory for Cancer Research, Frederick, Maryland, United States of America; Columbia University, UNITED STATES

## Abstract

To facilitate functional investigation of the role of NADPH oxidase 1 (NOX1) and associated reactive oxygen species in cancer cell signaling, we report herein the development and characterization of a novel mouse monoclonal antibody that specifically recognizes the C-terminal region of the NOX1 protein. The antibody was validated in stable NOX1 overexpression and knockout systems, and demonstrates wide applicability for Western blot analysis, confocal microscopy, flow cytometry, and immunohistochemistry. We employed our NOX1 antibody to characterize NOX1 expression in a panel of 30 human colorectal cancer cell lines, and correlated protein expression with NOX1 mRNA expression and superoxide production in a subset of these cells. Although a significant correlation between oncogenic RAS status and NOX1 mRNA levels could not be demonstrated in colon cancer cell lines, RAS mutational status did correlate with NOX1 expression in human colon cancer surgical specimens. Immunohistochemical analysis of a comprehensive set of tissue microarrays comprising over 1,200 formalin-fixed, paraffin-embedded tissue cores from human epithelial tumors and inflammatory disease confirmed that NOX1 is overexpressed in human colon and small intestinal adenocarcinomas, as well as adenomatous polyps, compared to adjacent, uninvolved intestinal mucosae. In contradistinction to prior studies, we did not find evidence of NOX1 overexpression at the protein level in tumors versus histologically normal tissues in prostate, lung, ovarian, or breast carcinomas. This study constitutes the most comprehensive histopathological characterization of NOX1 to date in cellular models of colon cancer and in normal and malignant human tissues using a thoroughly evaluated monoclonal antibody. It also further establishes NOX1 as a clinically relevant therapeutic target in colorectal and small intestinal cancer.

## Introduction

In mammalian cells without phagocytic potential, reactive oxygen species (ROS) are generated as a by-product of cellular processes such as mitochondrial respiration or xenobiotic metabolism, and are rapidly detoxified by antioxidant enzymes that include catalase, glutathione peroxidase, peroxiredoxin isoforms, and superoxide dismutase species to maintain redox homeostasis [1–3]. Robust empirical evidence has demonstrated that ROS can be produced in response to growth factor or cytokine stimulation, and that ROS are required to regulate signaling pathways involved in cell differentiation, growth, migration, and host defense [4–6]. Redox imbalance due to increased ROS production or reduced cellular antioxidant capacity may also lead to pathological oxidative stress that favors inflammation and promotes tumor initiation by inducing DNA damage, protein and lipid oxidation, and chromosomal instability [2, 7]. Inflammation also promotes tissue remodeling and angiogenesis, both of which are hallmarks of tumor cell invasiveness [8–10]. In addition to mitochondrial respiration, ROS, which are generally present at higher concentrations in cancer cells compared to their non-malignant counterparts [11, 12], are produced by a family of transmembrane flavoenzymes, the NADPH oxidases [1, 13–16]. The seven NOX isoforms (NOX1-5, DUOX1, and DUOX2) vary in their tissue distribution, activation mechanisms, and the identity of the ROS produced; activated NOX1, NOX2, NOX3, and NOX5 generate superoxide, while DUOX1-2, and constitutively active NOX4 produce hydrogen peroxide [17–23].

NOX1 is primarily found in normal colonic epithelium [17]; to date, expression of NOX1 has also been shown to be increased in small series of tissue specimens from patients with pre-malignant chronic inflammation of the colon and small intestine, as well as in patients with colon adenocarcinomas [24–29]. Unfortunately, advanced colorectal cancer (CRC) remains refractory to the vast majority of existing therapies [30]; and genetic heterogeneity renders the identification of molecular markers of CRC pathogenesis difficult, although the adverse effect of chronic inflammation in the bowel is well-known [8, 16, 31]. Despite empirical evidence that NOX1 is upregulated in certain CRC cell lines and in a modest number of human tumor specimens [32, 33], a detailed understanding of the prevalence and distribution of NOX1 in CRC is lacking. This is in part due to the paucity of good quality, specific NOX1 monoclonal antibodies with applicability to a wide variety of assays. Therefore, we initiated a series of experiments focused on elucidating the functional extent of NOX1 in colon cancer cells following the elaboration of a validated human antibody targeting NOX1.

We describe herein the development of a NOX1 mouse monoclonal antibody (mAb) designed to target the cytosolic portion of the NOX1 protein spanning the NADPH and FAD binding domains (residues 224–564). We demonstrate in our studies that this novel NOX1 mAb effectively detects NOX1 protein expression over a wide dynamic range, from a genetically-engineered cell clone that expresses large quantities of NOX1, to colon cancer cell lines with more modest endogenous NOX1 expression levels, and to a large array of human epithelial cancer specimens that vary dramatically in their expression of NOX1. Because our NOX1 mAb was evaluated by Western blot analysis, confocal microscopy, flow cytometry, and immunohistochemical (IHC) staining, these studies allowed the identification of relevant cellular models of colon cancer in which to study NOX1 biology. Indeed, we demonstrate in these experiments that the immunoreactivity of our NOX1 mAb correlated very well with PMA-mediated ROS production as measured by luminescence assay; we also demonstrate that the long form of NOX1, *NOX1-L*, is the predominant form of NOX1 that contributes to superoxide production in colon cancer cell lines. Contrary to previous investigations, we found that NOX1 expression did not significantly correlate with alterations in the RAS gene in these cell lines. Furthermore, we demonstrate by IHC that NOX1 staining intensity significantly differed between malignant colon and small intestinal tumors when compared to normal colonic and small intestinal mucosa. Finally, we found that unlike prior investigations performed with smaller numbers of patient tumor samples, enhanced NOX1 expression in common human epithelial malignancies, other than small and large bowel tumors, could not be demonstrated at the protein level to be enhanced compared to normal tissues from those organs. Taken together, these results support a role for NOX1 in the etiology of malignancies of the large and small bowel.

## Materials and methods

### Cell culture and transfection

All human colon tumor cell lines or other cell lines were purchased from the American Type Culture Collection (ATCC, Manassas, VA), except for the UACC-257 line, which was provided by the Developmental Therapeutics Program, National Cancer Institute (Bethesda, MD). The cell lines were propagated at 37°C with a humidified incubator in an atmosphere of 5% CO_2_ and 95% air. Cell culture medium contained 10% FBS (catalog no. 100–106, Gemini, West Sacramento, CA). HT-29 (ATCC catalog no. HTB-38) and HCT-116 cells (catalog no. CCL-247) were grown in McCoy’s A medium (catalog no. 12-688F, Lonza, Walkersville, MD). LoVo cells (catalog no. CCC-229) were grown in F-12K medium (catalog no. 30–2004, ATCC, Manassas, VA). T84 cells (catalog no. CCL-248) were cultured with DMEM:F-12 medium (catalog no. 30–2006, ATCC). Caco-2 (catalog no. HTB-37), LS174T (catalog no. CL-188), RKO (catalog no. CRL-2577), RKO-E6 (catalog no. CRL-2578), LS123 (catalog no. CCL-255), WiDr (catalog no. CCL-218), and CCD-18Co (catalog no. CRL-1459) cell lines were grown in EMEM (catalog no. 112-018-101, Quality Biological, Gaithersburg, MD). LS513 (catalog no. CRL-2134), LS1034 (catalog no. CRL-2158), HCT-8 (catalog no. CCL-244), COLO 201 (catalog no. CCL-224), COLO 205 (catalog no. CCL-222), COLO 320DM (catalog no. CCL-220), COLO 320HSR (catalog no. CCL-220-1), DLD-1 (catalog no. CCL-221), HCT-15 (catalog no. CCL-225), NCI-H508 (catalog no. CCL-253), BxPC-3 (catalog no. CRL-1687), and UACC-257 cell lines were cultured with RPMI-1640 (catalog no. HS30255.01, GE Healthcare Life Science, Logan, Utah). p53R (catalog no. JHU-56), SW1116 (catalog no. CCL-233), SW1417 (catalog no. CCL-238), SW403 (catalog no. CCL-230), SW48 (catalog no. CCL-231), SW480 (catalog no. CCL-228), SW620 (catalog no. CCL-227), SW837 (catalog no. CCL-235), and SW948 (catalog no. CCL-237) cells were grown in DMEM medium (catalog no. 112-014-101, Quality Biological, Gaithersburg, MD).

Stable clonal cell lines overexpressing NOX1 (HEK293-NOX1), NOX2 (HEK293-NOX2), or NOX4 (HEK293-NOX4), and UACC-257 melanoma cells stably overexpressing NOX5 were engineered in-house. The HEK293-NOX1 clone stably expressing NOX1/NOXA1/NOXO1 was initiated by transfection of pCMV-NOX1 plasmid (3 μg) using the Lonza system (Kit V, Program Q-001) [34]. The HEK293-NOX2 clone was obtained by transfection of Myc-DDK-tagged-NOX2 plasmid (4 μg) (#RC208997, Origene, Rockville, MD), and the HEK293-NOX4 clone was obtained by transfection of pCMV-MycDDK-HsNOX4 plasmid (4 μg) (#RC208007, Origene, Rockville, MD), using the Lonza system (Kit V, Program Q-001) [34, 35]. pcDNA3-HA-NOX5β plasmid (4 μg) (#AF325189, Origene, Rockville, MD) was transfected into parental UACC-257 cells using Lipofectamine 2000 (catalog no. 11668019, ThermoFisher Scientific, Rockford, IL) according to the manufacturer’s protocol to produce a stable NOX5-overexpressing UACC-257 clone [36]. An empty vector was used as control for each transfection. Resistant clones were selected with 700 μg/ml G418 (catalog no. 5005, Teknova, Hollister, CA), and single clones were then maintained under G418 selection. For gene silencing experiments, 1x10^6^ LS513 cells in log-phase were transfected with 5 nM NOX1 Silencer^®^ Select siRNA (catalog no. 4390817, Ambion, Austin, TX) or a Silencer^®^ Negative Control siRNA (catalog no. AM4635, Ambion, Austin, TX) using the Lonza system (Kit V, program Q-001, Walkersville, MD). After 72 h of transient transfection, silencing efficiency was analyzed by measuring NOX1 RNA level using TaqMan real-time PCR and protein level by Western blot analysis.

### Cloning, expression, and purification of a partial recombinant NOX1 protein

The sequence of the human transmembrane glycoprotein NOX1 (564 AA) was obtained from the NCBI database, and analysis revealed the existence of rare codons and hydrophobic regions that hindered the expression of the full NOX1 protein in BL21 (DE3) *E*. *coli* with different plasmid vector backbones. To circumvent this technical issue, a recombinant, codon-optimized, partial NOX1 protein was synthesized that contained C-terminus residues 224 to 564. This region encompasses the cytosolic NADPH and FAD binding domains of NOX1. Using a bioinformatic approach, we confirmed the high potential immunogenicity of the truncated recombinant NOX1 protein for antibody production (S1B Fig). The NADPH- and flavin-binding regions of NOX1 share less than 40% sequence identity with the paralogous region of NOX4, NOX5, DUOX1, and DUOX2. NOX2 and NOX3 share the largest proportion of identical residues with NOX1 in the region of interest with 61% sequence identity (S2 Fig). Taken together, these results supported the 224–564 residue region of human NOX1 protein as a favorable target to ensure antibody specificity. The 1,023 bp fragment corresponding to residues 224–564 was amplified and sub-cloned into a pET-30a(+) vector. The predicted molecular weight for the truncated recombinant NOX1 protein was ~41 kDa. The soluble fraction of induced BL21 lysate was treated with 1% SDS and 2M urea to dissolve inclusion bodies, before further purification by gel filtration, and detection by SDS-PAGE.

### Production of mouse monoclonal NOX1 antibody

The purified recombinant, truncated NOX1 protein containing a 341 carboxy-terminal amino acid immunogenic fragment corresponding to the 224–564 amino acid region of the human NOX1 enzyme was used as an immunogen for the preparation of monoclonal antibodies. The recombinant NOX1 protein was expressed in *Escherichia coli*, and purified protein was used as an antigen to immunize Balb/C mice for NOX1 monoclonal antibody production [28]. The detailed procedure for the antibody generation and ELISA screening was previously described by Antony *et al*. [36]. Thirty-two ascites producing clones were further evaluated by Western blot; clone 4 was chosen for use in the NOX1 antibody studies.

### RNA isolation and TaqMan real-time PCR

Total cellular RNA was isolated following the manufacture’s protocol using the RNeasy Mini Kit (catalog no. 74104, Qiagen, Valencia, CA). A 20-μl reaction system containing 2 μg of total RNA was employed for cDNA synthesis using SuperScript II reverse transcriptase (catalog no. 18080–044) and random primers (catalog no. 48190–011) from Invitrogen (Carlsbad, CA). The cDNA synthesis cycle was as follows: 25°C for 5 min, 42°C for 50 min, and 75°C for 5 min. Real-time RT-PCR was performed on a 384-well plate in a 20-μl reaction system containing 2 μl of 1:5 diluted cDNA as previously described [36, 37]. The human primers for NOX1 (assay ID Hs00246589_m1), NOXA1 (assay ID Hs00736699_m1), NOXO1, (assay ID Hs00376039_g1), NOX2 (assay ID Hs00166163_m1), NOX3 (assay ID Hs00210462_m1), NOX4 (assay ID Hs00276431_m1), NOX5 (assay ID Hs00225846_m1), DUOX1 (assay ID Hs00213694_m1), DUOXA1 (assay ID Hs00328806_m1), DUOX2 (assay ID Hs00204187_m1), DUOXA2 (assay ID Hs01595312_g1), RAC1 (assay ID Hs00251654_m1), RAC2 (assay ID Hs00427439_g1), p22^phox^ (assay ID Hs00164370_m1) human actin primer (assay ID Hs99999903_m1), and TaqMan Universal PCR mix (4304437) were purchased from Applied Biosystems (Foster City, CA). Triplicate determinations were performed for each gene of interest.

### Detection of *NOX1-S/NOX1-Lv* and *NOX1-L*

Two long form NOX1 isoforms have been described in the literature; namely, the full-length functional form *NOX1-L*, and an inactive smaller splice variant that lacks exon 11, referred to as *NOX1-S* or *NOX1-Lv* [28, 38]. In the present work, *NOX1-S* and *NOX1-Lv* were both used interchangeably to designate the same NOX1 variant. PCR primer sets corresponding to *NOX1-L* and *NOX1-S*/NOX1-Lv, 5’-GGGCTTTCGAACAATAT-3’ and 5’-CGAGGGCCACATAAGAAAA-3’, respectively, were designed and used to detect human NOX1 mRNA, which produces 502 (*NOX1-L*) and 355 (*NOX1-S*/*NOX1-Lv*) base pair amplicons [28]. The Taq DNA Polymerase kit (cat # 11304) was purchased from Invitrogen (Carlsbad, CA), and 2 μl cDNA was used for PCR reaction setup according to the manufacturer’s protocol. A β-actin primer (Integrated DNA Technologies, Coralville, IA) reaction was also setup as an internal control. Cycling conditions were as follows: 30 s at 95°C for initial denaturation; followed by 30 cycles of 30 sec 95°C denaturation, 30 s annealing at 55°C, and 1 min extension at 68°C; and fine extension at 68°C for 5 min. The PCR product (10 μl) was loaded onto 1.2% agarose gels, and electrophoresis was conducted at 100 V. Gel images were obtained using a ChemiDoc Touch Imaging System (Bio-RAD, Hercules, CA).

Untagged full length NOX1 (pCMV-*NOX1-L*) and untagged variant NOX1 (pCMV-*NOX1-Lv*) expression plasmids were generated by shuttling the full length and variant insert sequences from pCMV6-entry vectors (catalog no. PS10001, Origene, Rockville, MD) via SgfI (catalog no. R7103, Promega, Madison, WI) and MluI (catalog no. R0198S, New England Biolabs, Ipswich, MA) restriction enzyme sites in to the pCMV6-A-puromycin tagged cloning vector (catalog no. PS100025, Origene, Rockville, MD). All plasmids generated with inserts were verified by DNA sequencing. HEK293-vector control stably expressing pooled clones were initiated by transfection of HEK293 cells with appropriate plasmid (2 μg) using the Lonza system (Kit V, Program Q-001). An empty vector (pCMV6) was used as a control for the transfection. Samples were collected after 48 h for transient transfection samples, while resistant clones (pooled stable) were obtained after selection with 500 μg/ml puromycin. NOX1 expression was analyzed by measuring NOX1 RNA level using TaqMan real-time PCR and protein level by Western blot analysis.

### Preparation of whole-cell lysates and subcellular fractions

Subcellular fractions of parental LS513 cells, NOX1-overexpressing clone HEK293-NOX1 cells, and NOX1-silenced LS513 cells were prepared using the Qproteome cell compartment kit (catalog no. 37502, Qiagen, Valencia, CA). The detailed procedures have been described previously [35, 36]. Briefly, the cells in log-phase growth were washed with ice-cold PBS buffer, and sequentially extracted using four extraction buffers, and separated into cytosolic, membrane, nuclear, and cytoskeletal fractions according to the manufacturer’s instructions. Immunoblotting with heat shock protein 90 (HSP90) (catalog no. 4877), protein tyrosine phosphatase 1B (PTP1B) (catalog no. 5311), poly(ADP-ribose) polymerase PARP (catalog no. 9532), and vimentin (catalog no. 3390) antibodies specific for cytosolic, membrane, nuclear, and cytoskeleton proteins, respectively, were used to verify the isolation of each fraction. The antibodies were purchased from Cell Signaling Technology (Danvers, MA).

For whole-cell protein lysates, cells in log-phase growth were washed with ice-cold PBS, harvested, and lysed with 1X RIPA lysis buffer (catalog no. 20–188, Millipore, Temecula, CA) supplemented with protease inhibitor (catalog no. 05892970001, Roche, Indianapolis, IN) and phosphatase inhibitor (catalog no. 04906837001, Roche, Indianapolis, IN) cocktail tablets. Lysed cell mixture was centrifuged at 6000 RPM for 10 min at 4°C. Supernatant was collected, and protein concentration was determined using the BCA Protein Assay Kit (catalog no. 23225, Pierce Biotechnology, Rockford, IL); BSA was used as the standard.

### Western blot analysis

Protein lysates were obtained as described above. Lysates were mixed with an equal volume of 2X SDS protein gel loading buffer (catalog no. 351-082-661, Quality Biological Inc, Gaithersburg, MD), and 40 μg of protein—20 μg for NOX1-overexpressing HEK293 cells—were loaded onto a 4–20% Tris glycine gel (catalog no. EC6028, Invitrogen, Carlsbad, CA). Following gel separation, the proteins were transferred onto nitrocellulose membranes using the iBlot^™^ 2 gel transfer system (catalog no. IB23001, Invitrogen, Carlsbad, CA). Membranes were blocked in 1X TBST buffer containing 5% non-fat milk for 1 h at room temperature and incubated with primary antibody overnight at 4°C with slow rocking. Membranes were washed four times in 1X TBST buffer and incubated with a mouse or rabbit HPR-conjugated secondary antibody for 1 h at room temperature with shaking. SuperSignal West Pico Luminol/Enhancer solution (catalog no. 1856136, ThermoFisher Scientific, Rockford, IL) was used to visualize specific antigen-antibody binding.

### NOX1 immunodetection by confocal microscopy

5x10^3^ stable NOX1-overexpressing HEK293 cells (HEK293-NOX1), vector control cells (HEK293-vector), LS513, or HT-29 cells were seeded on Permanox 4-well chamber slides (catalog no. 177437; ThermoFisher Scientific, Rockford, IL). After 72 h, the cells were rinsed with PBS twice and fixed with 4% (w/v) paraformaldehyde for 10 min at room temperature. Cells were permeabilized with 0.1% Triton X-100 (v/v) for 5 min at room temperature. Following two washes with PBS, the cells were blocked with 5% BSA in PBS for 1 h at room temperature. After two further washes, the cells were incubated with the mouse monoclonal NOX1 antibody at 1:1000 dilution or with a mouse IgG (as a negative control) in 0.5% BSA overnight at 4°C. Following 3 washes with PBS, the cells were incubated with a secondary Alexa Fluor 488 goat anti-mouse antibody (catalog no. A-11029, ThermoFisher Scientific, Rockford, IL) at 1:1000 dilution with 1% BSA in PBS for 1 h. The cells were washed, and cell nuclei were stained and mounted in Vectashield with DAPI (catalog no. H-1200, Vector Laboratories, Inc., Burlingame, CA). Samples were then stored at −4°C in the dark. All images were obtained using the LSM 710 NLO confocal microscopy system, and the companion imaging software suite, Zeiss Efficient Navigation ZEN (Carl Zeiss Microscopy, LLC, White Plains, NY).

### Transient transfection in LS513 cells

To cross-validate the specificity of our novel NOX1 monoclonal antibody in a cell line that expresses both NOX1 and a different NOX isoform, 3x10^6^ LS513 cells in 8 ml RPMI-1640 buffer supplied with 10% FBS were added into a 10 cm dish containing 2 ml of transfection mix 2 ml Opti-MEM and 25 μl lipofectemine RNAiMAX mix (catalog no. 13778–150, Invitrogen, Carlsbad, CA) in presence or absence of 10 nM of Silencer® Select NOX1 siRNA (catalog no. 4392420, ID no. S25726 and S25727, Ambion, Austin, TX), Silencer® Select DUOX2 siRNA (catalog no. 4392422, Ambion, Austin, TX), or Silencer® Negative control siRNA (catalog no. 4390843, Ambion, Austin, TX), pre-incubated for 15 min at room temperature. After 24 h of incubation at 37°C and 5% CO_2_, 25 ng/ml each of IL-4 (catalog no. 204-IL-050, R&D Systems, Minneapolis, MN) and IL-17A (catalog no. 307-ILB-050, R&D Systems, Minneapolis, MN) were added to the cells to induce DUOX2 expression. The cells were incubated for an additional 24 h and then harvested for protein extraction. Western blot analysis was performed to detect NOX1 and DUOX2 expression in the LS513 samples.

### Immunodetection by flow cytometry

HEK293-vector and HEK293-NOX1 cells in log-phase growth were harvested, trypsinized, washed in cold PBS, and pelleted. The assay procedures were previously described by Antony *et al*. [36]. Briefly, following centrifugation the cell pellet was suspended and fixed with cold 2% (w/v) paraformaldehyde (catalog no. 15713-s, Electron Microscopy Sciences, Hatfield, PA) in PBS for 30 min on ice. The fixation solution was then removed by centrifugation, and the cell pellets were resuspended and permeabilized with 0.2% Tween 20 solution in PBS for 15 min in a 37°C water bath. Following centrifugation, 10^6^ permeabilized or non-permeabilized cells were either unlabeled with PBS or labeled with mouse IgG antibody (3 μg) or NOX1 specific mouse monoclonal antibody (3 μg) in heat-inactivated human AB serum for 30 min at 4°C in the dark. The cells were then washed twice with 0.2% Tween solution in PBS followed by incubation with secondary Alexa Fluor 488 goat anti-mouse antibody at 1:1500 dilution in heat-inactivated human AB serum for 20 min on ice in the dark. Cells were then centrifuged, washed twice, and suspended in PBS. Fluorescence intensity of the cells was measured on a FACScalibur cytometer (Becton Dickinson Biosciences, San Jose, CA), acquired using the data file acquisition program CellQuest (Becton Dickinson Biosciences, San Jose, CA), and analyzed using FlowJo^®^ software.

### Superoxide anion chemiluminescence assay

Superoxide anion (O_2_^•−^) production was detected using the luminol-based Superoxide Anion Assay Kit (catalog no. CS1000, Sigma-Aldrich, St. Louis, MO). Six cell lines with appreciable NOX1 mRNA expression (LS513, NCI-H508, LS174T, HT-29, WiDr, and HEK293-NOX1) and three cell lines with undetectable NOX1 expression (HCT-116, RKO, and SW620) were harvested in log-phase growth and counted. Following the manufacturer's instructions, cells were resuspended in assay medium at a density of 2x10^6^ cells/ml for HEK293-vector and HEK293-NOX1, and 10^7^ cells/ml for all other cell lines. One hundred microliter of each cell suspension was added per well in a 96-well plate. Before starting measurements, 100 μl of assay buffer containing 3 μl of luminol solution, 3 μl of enhancer solution was added to each well, in the presence or absence of 200 nM phorbol 12-myristate 13-acetate (PMA, catalog no. P8139, Sigma-Aldrich, St. Louis, MO) and 200 U/ml superoxide dismutase-polyethylene glycol from bovine erythrocytes (PEG-SOD, catalog no. S9549, Sigma-Aldrich, St. Louis, MO). The samples were mixed and placed immediately into a GloMax^®^ Microplate Luminometer (Promega BioSciences, San Luis Obispo, CA), and the luminescence was measured at 37°C during a 1.5–2 h period of observation. Every experiment was performed in triplicate.

### Determination of superoxide production by cytochrome c reduction

2x10^6^ cells per well of HEK293-NOX1, HEK293-vector control, LS513, or HT-29 cells were suspended in 200 μl of HBSS-HEPES buffer (catalog no. H8264, Sigma-Aldrich, St. Louis, MO; reference no. 15630–080, ThermoFisher Scientific, Rockford, IL) containing 100 μM cytochrome c partially acetylated from equine heart (catalog no. C4186, Sigma-Aldrich, St. Louis, MO), 200 nM PMA, and/or 200 U/ml PEG-SOD (catalog no. S9549, Sigma-Aldrich, St. Louis, MO). The change in optical density at 550 nm was quantified at 60 min using the known kinetics of cytochrome c reduction [39, 40]. To eliminate superoxide-independent cytochrome c reduction, the absorbance value with PEG-SOD was subtracted from the absorbance value with cytochrome c alone for each cell line system, before conversion to nmol superoxide produced per hour per 10^6^ cells using the method described by Bellavite *et al*. [41].

### DNA sequencing of RAS genes

Twenty-eight colon tumor cell lines in log-phase growth were harvested and washed twice with cold PBS; 1x10^7^ cells from each colon cancer line were collected and resuspended in 400 μl of PBS. The QIAamp DNA kit (catalog no. 51304, Qiagen, Gaithersburg, MD) was used for Genome DNA collection and purification for each cell line according to the manufacturer’s protocol. Following purification, DNA concentration and purity were measured on the Nanodrop ND-1000 apparatus (Nanodrop Technologies, Wilmington, DE). The DNA samples were then shipped to the CLIA Molecular Diagnostics Laboratory, Leidos Biomedical Research, Inc. (Frederick, MD) for detection of HRAS, KRAS, and NRAS mutations by Sanger sequencing. Transcribed RNA regions were analyzed and compared to the published gene sequences to identify variants.

### Data mining and bioinformatic analyses

For 62 bowel cancer cell lines of the Broad Institute Cancer Cell Line Encyclopedia (CCLE), processed RNA-seq gene expression data and germline-filtered mutation annotation format (MAF) files for NOX1, HRAS, KRAS, and NRAS were accessed and downloaded from the CCLE web portal (https://portals.broadinstitute.org/ccle) [42]. Sample-level, quantile-normalized RSEM (RNA-Seq by Expectation-Maximization) expression data and mutation annotation files for the TCGA colorectal adenocarcinoma dataset generated by the TCGA Research Network (http://cancergenome.nih.gov/) were accessed and downloaded through the Broad Institute’s Firehose (https://gdac.broadinstitute.org/). Normalized mRNA expression values were mean-centered, and unit variance scaling was applied to derive relative NOX1 expression in each dataset.

### Immunohistochemistry and tissue microarray

IHC staining was performed on 1,206 formalin-fixed, paraffin-embedded tissue microarray (TMA) blocks from US Biomax, Inc. These included: colon disease tissue arrays #BC05002, #BC051110, and #CO809a, small intestine tissue array #SM2081, breast invasive ductal carcinoma tissue array #BC08118, breast cancer tissue array #BR1505b, lung cancer tissue array #LC2085b, mid-advanced stage ovary cancer tissue array #OV8010, prostate cancer tissue array #PR2085b, and a tissue array of gastritis with intestinal metaplasia and gastric carcinoma #IC00011. The TMAs were deparaffinized in xylene and rehydrated with graded alcohol prior to antigen retrieval. Our NOX1 antibody developed in mouse was used. Slides were then counterstained with hematoxylin, dehydrated, and cover slipped. All samples were processed in parallel with a no-primary-antibody control to evaluate possible artefactual nonspecific staining from the secondary antibody. Isotype control staining was prepared with normal mouse IgG (BD Biosciences, San Jose, CA) at a comparable concentration to the primary antibody. The verification of staining performance was confirmed on a series of cancer tissue samples. In addition, a series of normal, non-tumor tissues were evaluated to establish immunoreactivity and assay specificity. Evaluation and comparison of staining on sections exposed to the primary and secondary antibodies were compared to negative control sections that were not exposed to the primary antibody. Each slide was digitally imaged using an Aperio ScanScope^®^. Tissues were scored as positively stained only if they exhibited a staining pattern with the primary antibody that was significantly different than that found when omitting the primary antibody. Those that did not demonstrate a significant difference between primary and omitting primary staining were graded as 0+ no stain and 0% cells stained. Tissues that demonstrated a significant difference between the two conditions were graded as follows. The assay was interpreted with a scoring system of 0+, 1+, 2+, and 3+ for staining intensity corresponding to negative, weak, moderate, and strong NOX1 staining. The percentage of stained tumor/lesion cells (distribution) was estimated for each case, where 0% to <10% was considered negative.

### Statistical analyses

All data are expressed as means ± standard deviation (SD) of at least 3 independent experiments. Statistical differences between control and treated cell lines were assessed with the non-parametric Mann-Whitney test. For the comparison of NOX1 mRNA expression level between sample groups with different RAS mutation status, the Mann-Whitney test was used. Significance levels were designated as *p<0.05, **p<0.01, and ***p<0.001 throughout.

## Results

### Production and characterization of a monoclonal antibody against NOX1

**Fig 1:**
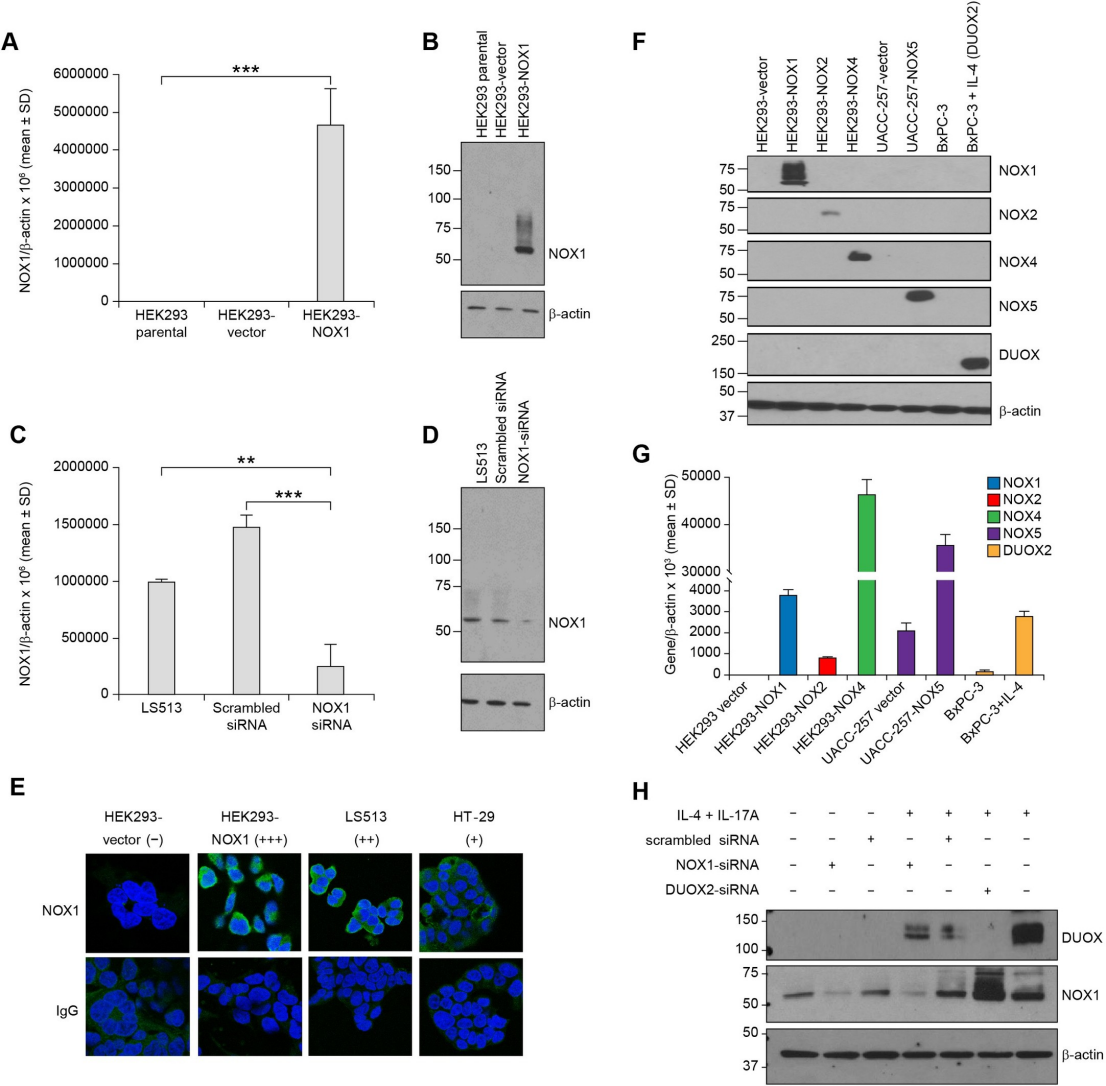
Characterization and validation of the novel NOX1 mouse monoclonal antibody. **(A, B)** HEK293 cells were stably transfected with the pCMV-NOX1 plasmid or an empty vector and selected with G418. NOX1 overexpression was confirmed **(A)** at the mRNA level by RT-PCR (***p<0.001 vs. untransfected cells), and **(B)** at the protein level by Western blot analysis. **(C, D)** Transient knockdown of NOX1 expression in the colon cancer cell line LS513 with a scrambled control or a NOX1-specific siRNA. **(C)** A 4-fold decrease in NOX1 expression compared to parental (**p<0.01) and 6-fold decrease compared to cells transfected with scrambled siRNA (***p<0.001) was noted after 72 h at the mRNA level by RT-PCR. NOX1 mRNA level is given relative to β-actin. Data represent mean ± SD for at least 3 independent experiments. **(D)** Western blot analysis confirmed the NOX1 decrease at the protein level. **(E)** Immunodetection of NOX1 in HEK293-NOX1 and HEK293-vector control, LS513, and HT-29 cells by confocal microscopy. The cells were immunostained with NOX1 mouse mAb (green). Cell nuclei were stained with 4′,6-diamidino-2-phenylindole (DAPI; blue), and mouse anti-IgG was used as a negative control. Digital images were taken at 63X magnification. NOX1 protein expression is qualitatively labeled as -, no expression; +, relatively low expression; ++, relatively higher expression; +++, highest expression. **(F)** To demonstrate a lack of cross-reactivity of the NOX1 antibody with other NOX isoforms, protein levels were measured by Western blot analysis for HEK293 cells stably transfected with the pCMV-NOX1 plasmid, the Myc-DDK-tagged-NOX2 plasmid, the pCMV-MycDDK-HsNOX4 plasmid, or an empty vector; UACC-257-vector and UACC-257-NOX5 stable overexpressing clones; and BxPC-3 cells with or without IL-4 stimulation (25 ng/ml for 24 h). The antibodies used to detect the NOX isoforms are listed in the Materials and Methods section. **(G)** RNA levels of the NOX isoforms were measured by RT-PCR. **(H)** NOX1 detection in a real-world system, LS513 colon cancer cells, that express both NOX1 and DUOX2 following 24-h incubation with IL-4 plus IL-17A, and treated with either a NOX1- or DUOX2-specific small interfering RNA (siRNA), or a nonspecific scrambled siRNA.

Our mouse monoclonal antibody (mAb) was raised against a truncated recombinant peptide (residues 224–564) of the NOX1 protein (S1 Fig). The set of amino acids contained in this region of the NOX1 protein share no more than 61% sequence identity with other NOX proteins (S2 Fig). After 4 rounds of immunization in Balb/C mice and 2 rounds of screening, supernatant from 3 ELISA-positive stable hybridoma clones (#4, #22, and #55, S1 Table) was further evaluated for NOX1 protein detection by Western blot. Following purification by peptide affinity column, clone 4 was used for the present study. The NOX1 antibody was validated using HEK293 cells genetically engineered to overexpress NOX1/NOXA1/NOXO1 (HEK293-NOX1) or an empty vector control. Significant NOX1 expression was detected in the NOX1-transfected cells by quantitative RT-PCR (***p<0.001 vs. untransfected, Fig 1A). Western blot analysis of the HEK293-NOX1 clone revealed a reactive band at ~65 kDa, consistent with the known molecular weight of NOX1, that was not present in untransfected and vector-transfected clone (Fig 1B). Furthermore, in the human colon cancer cell line LS513, transfection with a NOX1-specific siRNA reduced endogenous NOX1 mRNA expression by ≥75% (**p<0.01 vs. untransfected and ***p<0.001 vs. cells transfected with scrambled siRNA, Fig 1C); NOX1 protein expression was also decreased by NOX1-specific siRNA as indicated by the decreased intensity of the immunoreactive band at 65 kDa (Fig 1D). The specificity of our novel mouse mAb was further demonstrated by its lack of cross-reactivity with NOX1 homologs NOX2, NOX4, NOX5, and DUOX2 in relevant cell lines and systems: HEK293 clones stably overexpressing NOX1, NOX2, NOX4, or NOX5, and DUOX2-expressing BxPC-3 pancreatic cancer cells untreated or treated with 25 ng/ml IL-4 for 24 h (Fig 1F). The expression of the different NOX isoforms was confirmed by qPCR (Fig 1G). Furthermore, in the LS513 cell line in which two different NOX isoforms are expressed upon stimulation with IL-4 plus IL-17A, our NOX1 mAb specifically recognized NOX1, and did not recognize DUOX2 (Fig 1H). The NOX1 band was distinctly reduced in LS513 cells transiently transfected with NOX1-siRNA (lanes 2 and 4); the NOX1 signal remained with transient silencing of DUOX2 in LS513 cells treated with IL-4 plus IL-17A (lane 6), a cytokine combination that induces DUOX2 expression in these cells (lane 7). The lack of specificity of the novel NOX1 mAb for DUOX2 is expected, considering that in the relevant region of the NOX proteins, NOX1 and DUOX2 only share 30% sequence identity (S2 Fig).

### Validation of the NOX1 mAb for confocal microscopy, Western blot analysis, and flow cytometry

Permeabilized HEK293-NOX1 clone and HEK293-vector controls, LS513, and HT-29 cells were stained with the NOX1 mouse mAb (1:1000) and labeled with Alexa Fluor 488 goat anti-mouse secondary antibody (green fluorescence) for NOX1 detection by confocal microscopy (Fig 1E). Nuclei were stained with DAPI (blue), and mouse IgG mAb served as a negative control. HEK293-NOX1 overexpressing cells presented primarily cytoplasmic NOX1 staining with perinuclear enhancement. The subcellular localization of NOX1 was further investigated with cell fractionation studies. HEK293-NOX1 cell lysate was separated into cytosolic (F1), membrane (F2), nuclear (F3), and cytoskeletal fractions (F4). Adequate fractionation was confirmed with the presence of the following organelle markers: HSP90 (cytosol), Na/K ATPase (membrane), lamin A/C (nucleus), and vimentin (cytoskeleton) in fractions F1-F4, respectively. Cell fractionation revealed that NOX1 protein was localized in the pooled membrane fraction of HEK293-NOX1 clones, as indicated by the immunoreactive band at 65 kDa (S3A Fig). In LS513 colon cancer cells that endogenously express NOX1, the NOX1 band was also detected in lysate from the membrane fraction (S3B Fig, left), and the intensity of the immunoreactive band was strongly attenuated in LS513 cells transfected with NOX1 siRNA (S3B Fig, right). These data confirm the membrane localization of the NOX1 protein, in both recombinant and endogenous systems.

We also interrogated the binding of our NOX1 mAb to native antigen by flow cytometry in permeabilized (S4A Fig) and non-permeabilized (intact) HEK293-NOX1 clones (S4B Fig). Permeabilized HEK293-NOX1 cells demonstrated substantial NOX1 mAb bound compared to HEK293-vector control cells (S4A Fig, dark green and orange lines, respectively). This effect was not detected when comparing non-permeabilized HEK293-NOX1 and HEK293-vector control cells (S4B Fig), confirming that the epitope recognized by our NOX1 mAb is in the intracellular portion of the NOX1 protein, consistent with its design against the conserved C-terminus of NOX1 (residues 224–564).

### Functional NOX1 expression in colon cancer cell lines

**Fig 2:**
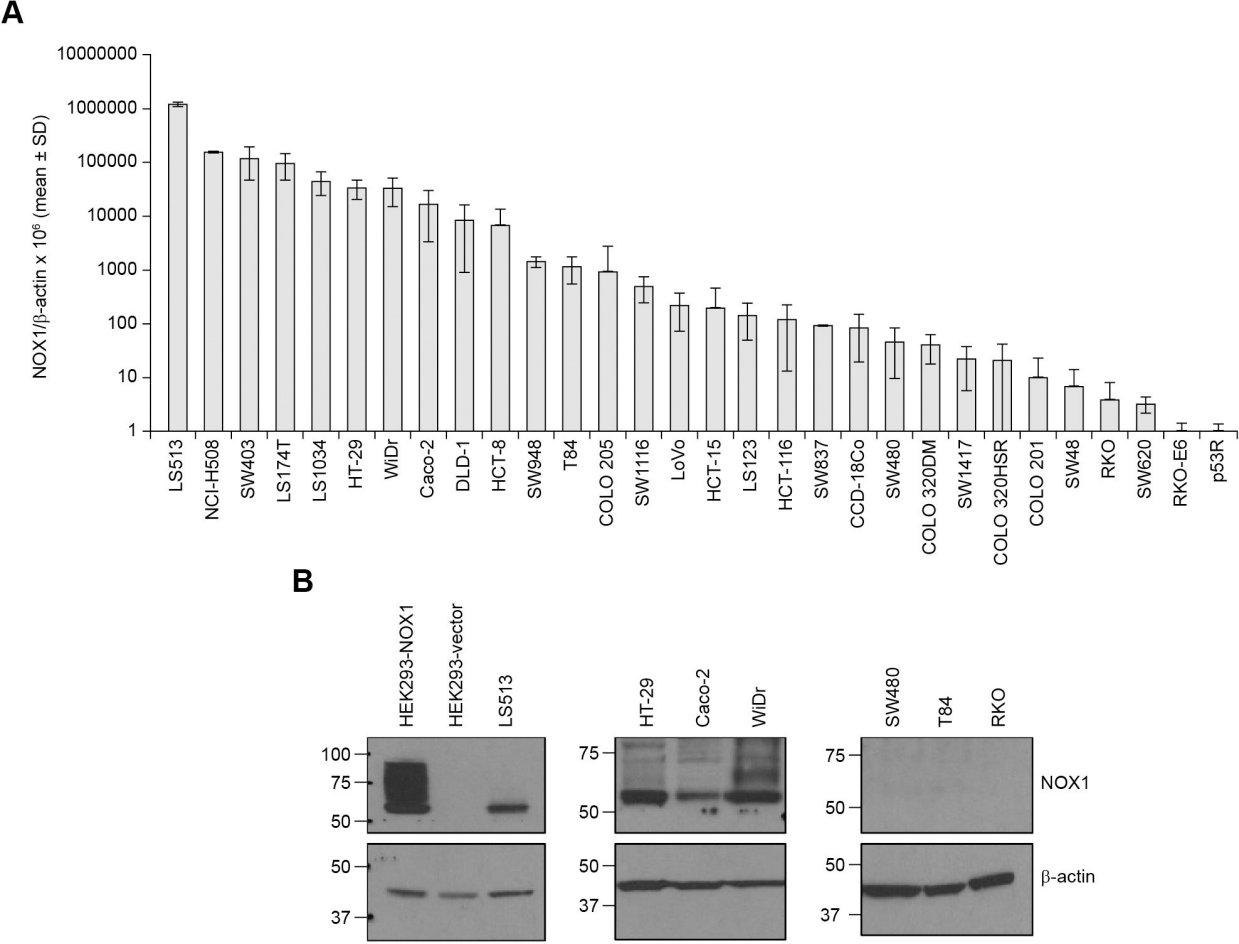
Detection of NOX1 in human colon cancer cell lines. **(A)** NOX1 expression was evaluated in a series of 30 human colon cancer cell lines obtained from the American Type Culture Collection. NOX1 mRNA level is given relative to β-actin. Data represent mean ± SD for at least 3 independent experiments. **(B)** NOX1 expression determined in parallel experiments for a panel of 7 colon cancer cell lines plus HEK293-NOX1 and HEK293-vector control at the protein level by Western blot analysis (2-min exposure).

**Fig 3:**
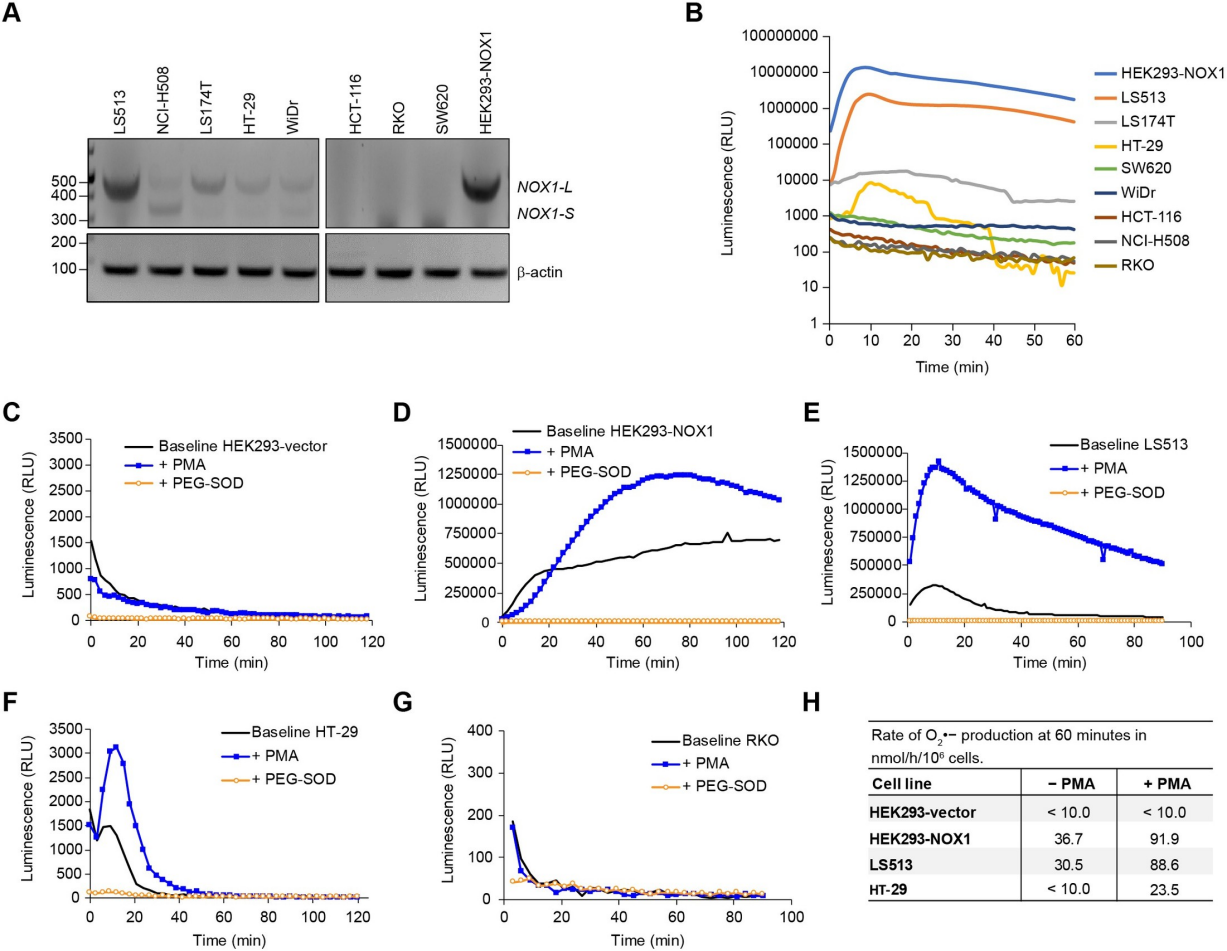
Functional expression of NOX1 isoforms in human colon cancer cell lines. **(A)** RT-PCR and subsequent DNA gel analyses were performed to detect *NOX1-L* and *NOX1-S*/*NOX1-Lv* in a subset of 8 colon cancer cell lines plus HEK293-NOX1. **(B)** The subset of cell lines was evaluated for PMA-stimulated superoxide production by luminescence assay. **(C-G)** Basal and PMA-stimulated superoxide production were also measured in **(C)** HEK293-vector control, **(D)** HEK293-NOX1, **(E)** LS513, **(F)** HT-29, and **(G)** RKO cells using the luminol assay. Superoxide dismutase-polyethylene glycol (PEG-SOD, 200 U/ml) was used to confirm superoxide production, with measurements taken every 2 min for up to 120 min. **(H)** Mean rate of superoxide production at 60 min in nmol/h/10^6^ cells. 2X10^6^ cells per well were suspended in 200 μl of HBSS-HEPES containing 100 μM acetylated cytochrome c with or without 200 nM PMA and/or 200 U/ml PEG-SOD. The change in optical density at 550 nm was quantified using the kinetics of cytochrome c reduction. To eliminate superoxide-independent cytochrome c reduction, the absorbance value with PEG-SOD was subtracted from the absorbance value with cytochrome c alone for each cell line system.

To develop model systems for examination of NOX1 functionality in colon cancer, we evaluated the range of NOX1 mRNA expression across a panel of 30 human CRC cell lines (Fig 2A). We found that one third of the cell lines demonstrated NOX1/β-actin ratios (x10^6^ as assessed by RT-PCR) ≥ 5000, a level that generally correlated with detectable NOX1 mRNA expression by Northern blot analysis in our previous experiments with cell lines expressing NOX1 [32]. As shown in Table 1, in addition to NOX1, both NOXA1 and NOXO1 were also expressed in these cell lines together with p22^phox^, providing, in theory, all the necessary components for a functional NOX1 complex. Furthermore, we found that except for low amounts of NOX2 in WiDr cells and DUOX2 in NCI-H508 cells, NOX1 was the major NOX isoform expressed at the mRNA level in the CRC lines we examined. Our validated NOX1 mAb was then employed to assess the expression level of NOX1 protein in a panel of 7 human colon cancer cell lines, as well as in the HEK293-NOX1 stable overexpressing clone and HEK293-vector control cells (Fig 2B). Western blot analysis of NOX1 protein expression was largely concordant with RNA levels evaluated by quantitative RT-PCR (Fig 2A), demonstrating NOX1 protein expression in HEK293-NOX1, LS513, HT-29, Caco-2, and WiDr; but not in HEK293-vector, SW480, T84, and RKO cells. At both the RNA and protein levels, LS513 cells demonstrated the highest amount of NOX1 expression after the HEK293-NOX1-overexpression system. Because presence of NOX1 mRNA and protein are not sufficient for NOX1 catalytic activity, we next examined the expression of the 2 known isoforms of NOX1—full-length functional *NOX1-L* and the inactive shorter splice-variant *NOX1-S* (also termed *NOX1-Lv*) that lacks exon 11 within the NADPH binding domain [28, 38]—in a subset of 9 cell lines: 8 colon cancer cell lines and the HEK293-NOX1 clone using two distinct PCR primers. The truncated isoform *NOX1-S*/*NOX1-Lv* was detected as the primary endogenous NOX1 isoform in NCI-H508 cells (Fig 3A). Moreover, we confirmed using HEK293 cells transiently and stably transfected with the inactive full-length isoform lacking exon 11, *NOX1-Lv*, that our novel NOX1 mAb specifically recognizes the functional *NOX1-L* isoform, but not the inactive *NOX1-Lv* variant by Western blot (S5B Fig, lanes 3 and 4 vs. lines 5 and 6) and by immunofluorescence (Fig 1E HEK293-NOX1 vs. S5C Fig HEK293-pCMV-*NOX1-Lv*).

**Table 1:** Expression of NOX isoforms and accessory molecules in human colorectal cancer cell lines.

*Cell line*	*NOX1*	*NOX2*	*NOX3*	*NOX4*	*NOX5*	*DUOX1*	*DUOX2*	*DUOXA2*	*NOXA1*	*NOXO1*	*Rac1*	*Rac2*	*p22*^*phox*^
LS513	1,211,451	4,343	ND	53	ND	ND	692	1,978	10,148	47,176	107,039	3,880	193,347
NCI-H508	154,446	2,782	ND	291	ND	61	8,799	405	2,949	6,829	117,176	10,294	299,644
SW403	120,303	418	ND	ND	11	2	30	ND	12,707	25,684	118,309	9,316	118,556
LS174T	96,065	1,562	ND	1	ND	8	28	ND	50,550	38,454	63,823	6,221	106,911
LS1034	45,194	1,586	ND	ND	26	377	15	ND	23,394	45,339	151,210	3,360	161,529
HT-29	33,697	491	ND	1	1	51	14	2	15,636	13,182	328,068	10,830	156,150
WiDR	33,513	18,123	ND	ND	0	31	5	1	11,885	3,572	142,890	1,139	53,446
Caco-2	16,975	20	6	3	13	1	585	42	4,583	949	153,771	36	81,943
DLD-1	8,546	594	ND	0	2	0	16	5	12,597	464	57,045	5,661	94,236
HCT-8	6,748	771	ND	ND	86	1	128		36,172	2,971	90,411	19,311	202,771
SW948	1,437	2,176	ND	6	ND	12	17	ND	28,722	12,386	163,247	3,922	89,333
T84	1,157	1,507	ND	ND	1	22	19	ND	20,451	2,671	119,087	8,911	66,810
COLO 205	931	1,291	ND	ND	19	6	7	1	7,695	3,349	12,283	1,866	15,496
SW1116	494	1,235	3	7	14	175	397	ND	40,999	10,956	225,033	4,132	94,088
LoVo	219	803	38	0	3	1	66	10	15,154	21,600	119,096	1,877	79,537
HCT-15	198	116	ND	0	0	0	2	0	17,993	1,155	41,611	9,306	85,216
LS123	144	1,198	25	ND	3	11	217	ND	2,105	1,094	68,604	1,701	19,269
HCT-116	118	571	ND	1	47	171	67	ND	17,367	712	63,656	20,240	91,143
SW837	95	2,103	ND	ND	ND	ND	ND	ND	32,104	16,093	60,891	5,609	137,725
CCD-18Co	85	12	ND	876	ND	2	1	ND	136	104	7,139	936	23,703
SW480	47	2,459	2	ND	26	ND	197	ND	4,276	2,132	70,716	2,960	56,281
COLO 320DM	40	478	36	0	1	0	2	1	6,423	40,674	76,221	18,679	91,145
SW1417	22	925	84	49	33	47	74	ND	27,318	42,325	135,057	3,245	60,670
COLO 320HRS	21	2,635	347	1	1	0	12	1	9,685	121	4,029	22	20,292
COLO 201	10	40	ND	0	1	6	5	4	5,817	29,118	100,998	25,409	118,595
SW48	7	1,286	1	0	2	0	1	1	15,932	737	63,412	1,332	58,438
RKO	4	206	ND	ND	ND	ND	2	ND	7,826	294	67,308	5,881	74,170
SW620	3	134	17	ND	7	10	5	ND	7,419	348	73,886	3,721	37,105
RKO-E6	1	436	ND	ND	ND	ND	7	ND	3,759	332	44,301	9,098	43,470
p53R	1	138	ND	ND	ND	ND	1	ND	317	71	43,545	5,407	34,780

Expression levels are shown as the mean ratio of the NOX isoform or accessory molecule to β-actin x 10^6^_;_ experiments were performed in triplicate. ND, not detectable.

Furthermore, as we have previously demonstrated, WiDr cells contain both *NOX1-S*/*NOX1-Lv* and *NOX1-L* mRNA (Fig 3A and [28]). In those studies and herein, the amount of *NOX1-L* was insufficient to produce enzymatically active NOX1. In support of these observations, we found no measurable PMA-stimulated luminescence in NCI-H508 cells and WiDr cells (Fig 3B, dark gray line and dark blue line, respectively), despite appreciable NOX1 mRNA expression (~155k NOX1/β-actin × 10^6^ for NCI-H508 and ~34k NOX1/β-actin × 10^6^ for WiDr, Fig 2A). In contrast, LS513, LS174T, HT-29, and HEK293-NOX1 expressed the active, full-length *NOX1-L* isoform (Fig 3A). The activity of NOX1 in these cells was assessed by luminescence assay to detect basal and PMA-dependent production of superoxide (Fig 3B–3G). While we observed basal superoxide production in NOX1-expressing cells, PMA enhanced superoxide-dependent luminescence in HEK293-NOX1 clones (peak chemiluminescence was increased by 1.6-fold), LS513 (+4.4-fold), and HT-29 (+1.7-fold), as illustrated in Fig 3D, 3E and 3F; but not in negative control cell lines HEK293-vector control and RKO (Fig 3C and 3G). The identity of the NOX1-associated radical species measured in these cell lines with the luminol assay was confirmed to be superoxide, as superoxide dismutase-polyethylene glycol (PEG-SOD) abrogated the luminescent signal in NOX1-expressing cells, but not in HEK293-vector control and RKO cells. In order to confirm the baseline and PMA-dependent production of superoxide in HEK293 cells overexpressing NOX1, and in HT-29 and LS513 colon cancer cell lines, a widely used assay that relies on measuring the reduction of acetyl-cytochrome c was also employed [43–45]. PEG-SOD was used as a control to suppress measured superoxide in these cell lines. The empirically-determined rate of superoxide production at 60 min in these cells, summarized in Fig 3H, was concordant with the results displayed in Fig 3B. As expected, PMA stimulation markedly enhanced superoxide production in the three NOX1-expressing cellular systems, but not in the HEK293-vector control; and superoxide production rate in LS513 cells was comparable to that of the engineered HEK293-NOX1 system. Taken together, these studies demonstrate that although mRNA and protein expression are not sufficient for NOX1 activity, we have identified multiple colon cancer cell lines in which NOX1 was functional, including, LS513, LS174T, HT-29, and HEK293-NOX1 cells; thus these cells constitute pertinent model systems with which to study NOX1 tumor biology. Furthermore, we have established that our NOX1 antibody specifically recognizes functional full-length NOX1.

### NOX1 and oncogenic RAS in human colon cancer cell lines

**Fig 4:**
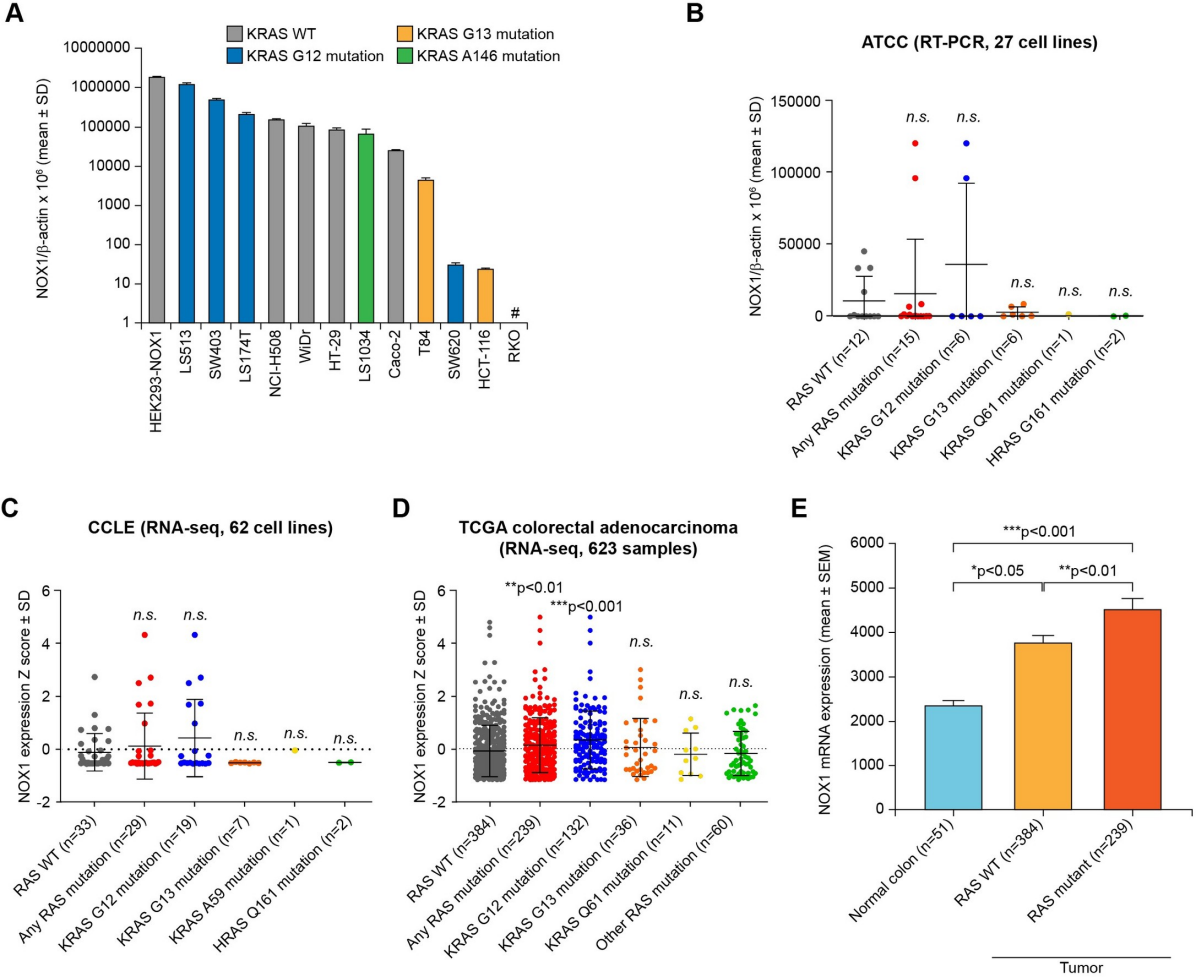
Expression of NOX1 and RAS mutation status in colon cancer cell lines and human colorectal tumor specimens. **(A)** NOX1 expression determined for a panel of 12 colon cancer cell lines plus HEK293-NOX1 at the mRNA level by qRT-PCR. NOX1 mRNA level is given relative to β-actin. Data represent mean ± SD for at least 3 independent experiments. Bars are color-coded according to KRAS mutation status. #, the RKO cell line is KRAS WT. **(B)** NOX1 expression was evaluated in a panel of 27 human colorectal cell lines at the mRNA level by quantitative real time PCR and is displayed relative to β-actin. Data represent mean ± SD for at least 3 independent experiments. **(C, D)** Relative NOX1 mRNA expression Z score in **(C)** 62 cell lines of the Cancer Cell Line Encyclopedia (CCLE) and **(D)** 623 colorectal adenocarcinoma specimens from The Cancer Genome Atlas (TCGA) stratified according to RAS mutation status. Each dot represents a cell line or tumor sample and the coloring corresponds to the RAS mutation status. **(E)** Mean NOX1 mRNA expression in 51 normal colorectal tissues from TCGA colorectal cohort was compared to expression in the 623 colorectal adenocarcinoma tumor samples from the same cohort shown in **(D)**, divided into RAS WT (n = 384) and RAS mutant (n = 239) groups. Data are ± SEM. Statistical significance for RAS WT vs. RAS mutant subcategories by the Mann-Whitney test: *p<0.05, **p<0.01, ***p<0.001; *n*.*s*. denotes the absence of statistical significance.

To investigate previous reports that superoxide-producing NOX1 is functionally required for RAS oncogene transformation [46], we initially examined the mutational status of KRAS in 12 human CRC cell lines that express NOX1 (Fig 4A). Observing no obvious correlation in this initial data set between RAS mutation status and NOX1 overexpression (Fig 4A), 27 colon cancer cell lines from the ATCC, 62 CRC cell lines from the Cancer Cell Line Encyclopedia (CCLE), and 623 colorectal tumor samples plus 51 normal colon specimens from The Cancer Genome Atlas (TCGA) were subsequently evaluated. For the CCLE and TCGA samples, RAS mutations were extracted from the respective open-source databases. For the 27 bowel cell lines from the ATCC, HRAS, KRAS, and NRAS mutations were assessed by Sanger sequencing at the NCI and corroborated using references from the literature (Table 2). In all 3 datasets, most RAS mutations occurred in the KRAS gene. We again found that NOX1 expression was not significantly overexpressed in KRAS mutant cell lines of the ATCC and CCLE compared to KRAS wild type cell lines (Fig 4B and 4C). In contrast, NOX1 mRNA expression was significantly overexpressed in RAS mutated TCGA CRC samples compared to RAS WT samples (**p<0.01, Fig 4D), although marginally (<1.4-fold). However, a statistically significant difference was found between NOX1 mRNA expression in normal colon tissue (n = 51) and malignant colorectal samples (RAS WT, n = 384, 1.6-fold NOX1 increase vs. normal colon, *p<0.05; RAS mutant, n = 239, 1.9-fold NOX1 increase vs. normal colon, ***p<0.001) from the TCGA (Fig 4E). However, there was no significant correlation in the ATCC, CCLE, or TCGA datasets between the mRNA expression levels of NOX1 and KRAS (S2 Table).

**Table 2:** RAS mutation status and NOX1 mRNA expression in 28 colon cell lines from the ATCC.

*Status*	*ATCC cell line*	*RAS mutation*	*NOX1/β-actin × 10*^*6*^	*Origin*	*Corroboration in literature*
	SW403	KRAS G12V	120303	Colorectal adenocarcinoma	[47–50]
	SW480	KRAS G12V	47	Colorectal adenocarcinoma	[47, 49–51]
	SW620	KRAS G12V	3	Colorectal adenocarcinoma	[47, 49–51]
	LS174T	KRAS G12D	96065	Colorectal adenocarcinoma	[49–51]
	LS123	KRAS G12S	144	Colorectal adenocarcinoma	[49]
	SW837	KRAS G12C	95	Colorectal adenocarcinoma	[47–50]
	DLD-1	KRAS G13D	8546	Colorectal adenocarcinoma	[50, 51]
	HCT-8	KRAS G13D	6748	Colorectal adenocarcinoma	[48]
	T84	KRAS G13D	1157	Colorectal carcinoma	[47–49]
	LoVo	KRAS G13D	219	Colorectal adenocarcinoma	[47–51]
	HCT-15	KRAS G13D	198	Colorectal adenocarcinoma	[47–51]
	HCT-116	KRAS G13D	118	Colorectal carcinoma	[47–51]
	SW948	KRAS Q61L	1437	Colorectal adenocarcinoma	[48–51]
HRAS mutant CRC	SW1116	HRAS G161R	494	Colorectal adenocarcinoma	KRAS G12A [48–51]
	SW48	HRAS G161R	7	Colorectal adenocarcinoma	KRAS WT [48–51]
RAS WT CRC	HT-29	RAS WT	33697	Colorectal adenocarcinoma	[48–51]
	WiDr	RAS WT	33513	Colorectal adenocarcinoma	[50, 51]
	Caco-2	RAS WT	16975	Colorectal adenocarcinoma	[48, 50, 51]
	COLO 201	RAS WT	10	Colorectal adenocarcinoma	[48]
	COLO 205	RAS WT	931	Colorectal adenocarcinoma	[48–50]
	COLO 320HSR	RAS WT	21	Colorectal adenocarcinoma	[49–51]
	COLO 320DM	RAS WT	40	Colorectal adenocarcinoma	[48, 50, 51]
	LS1034	RAS WT	45194	Colorectal carcinoma	KRAS A146T [49–51]
	SW1417	RAS WT	22	Colorectal adenocarcinoma	[48, 49]
	RKO	RAS WT	4	Colon carcinoma	[48–51]
	RKO-E6	RAS WT	1	Derived from RKO; disrupted p53 function	[48–51]
	p53R	RAS WT	1	Derived from RKO	[52]
RAS WT normal colon	CCD-18Co	RAS WT	85	Normal colon fibroblast	[53]

Mutations were identified by Sanger sequencing, and NOX1 mRNA level was measured by PCR and normalized to β-actin. CRC, colorectal cancer.

### Expression of NOX1 protein across human epithelial cancers and normal tissues

**Fig 5:**
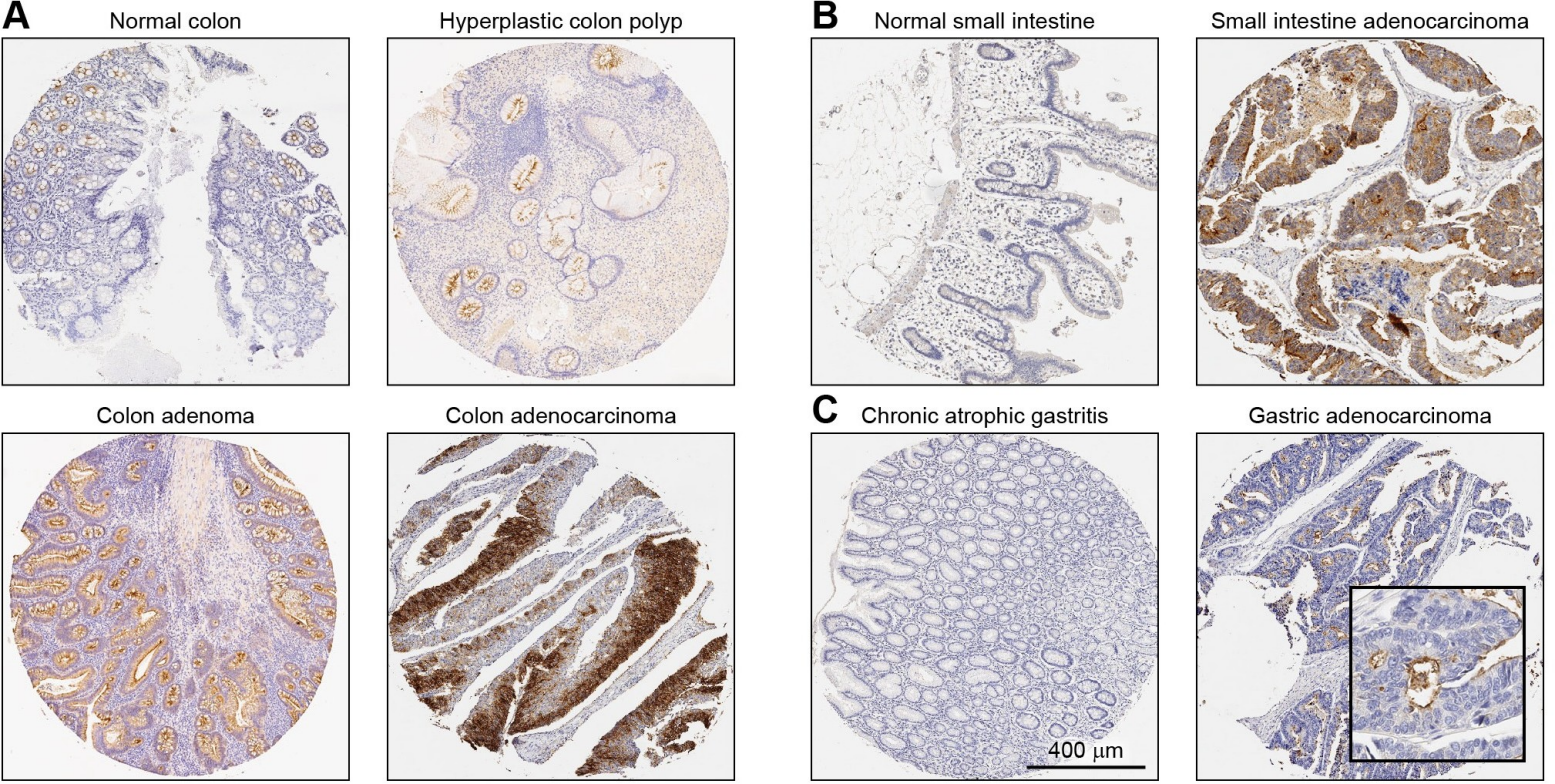
Immunohistochemical detection of NOX1 protein in tumors and corresponding non-neoplastic tissue of the same origin. Representative tumors/lesions and normal/non-neoplastic tissue pairs from a tissue microarray are shown. **(A)** Normal and abnormal colon, **(B)** normal and malignant small intestine, and **(C)** chronic atrophic gastritis and malignant gastric tissue. All images were taken at 10X digital magnification. The scale bar represents 400 μm.

The expression level of NOX1 protein was assessed with our mAb using multi-tumor tissue microarrays containing normal, benign, and malignant tissue samples of various anatomical origins by immunohistochemical (IHC) staining to establish which malignancies are associated with NOX1 overexpression (Fig 5). NOX1 staining intensity was assessed by a pathologist using the following scoring system: 0 (negative/no or blush staining), 1+ (weak), 2+ (moderate), and 3+ (strong). The percentage of stained tumor cells was estimated for each case. The distribution of positive NOX1 staining by tumor type is presented in Table 3; positive staining was compared between abnormal/malignant and non-malignant tissue by the *X*^*2*^ statistical test. Less than 5% of patients with prostate, ovary, lung, or breast carcinoma were high NOX1 expressers (Table 3). In contrast, our analyses revealed that the distribution of NOX1 staining was significantly different between normal colon mucosa and colon lesions (***p<0.001 for colon carcinomas and adenomas; *p<0.05 for colon polyps), as well as in small intestinal carcinomas relative to normal small intestinal tissue (*p<0.05, Table 3).

**Table 3:** NOX1 protein expression in human malignant and benign tissue.

Organ	Pathologic diagnosis	Unstained, unscored 0+ (No. [%])	Low expressers 1+ (No. [%])	High expressers 2+, 3+ (No. [%])	*P* value^1^ (statistical significance)	% cells positive			
						0‒9%	10‒24%	25‒49%	≥50%
Colon	Colon carcinoma	43 [29]	52 [36]	51 [35]	0.0 (***p)	29	10	12	49
	Colon adenoma	2 [6]	14 [44]	16 [50]	0.0004 (***p)	6	3	0	91
	Colon polyp	1 [8]	8 [67]	3 [25]	0.0314 (*p)	8	0	17	75
	Inflammatory lesions	0 [0]	11 [85]	2 [15]	0.0853 (*n*.*s*.)	0	8	8	85
	Normal colon	0 [0]	18 [100]	0 [0]		0	0	44	56
Small intestine	Small intestine carcinoma	56 [51]	37 [34]	17 [15]	0.0137 (*p)	51	20	12	17
	Small intestine inflammatory lesions	30 [88]	3 [9]	1 [3]	0.1562 (*n*.*s*.)	88	0	6	6
	Normal small intestine	25 [76]	8 [24]	0 [0]		76	0	12	12
Prostate	Prostate carcinoma	180 [98]	0 [0]	3 [2]	0.7328 (*n*.*s*.)	98	0	1	1
	Normal prostate	7 [100]	0 [0]	0 [0]		100	0	0	0
Ovary	Ovarian carcinoma	155 [95]	6 [4]	2 [1]	0.9497 (*n*.*s*.)	95	0	2	2
	Normal ovarian tissue adjacent to cancer	2 [100]	0 [0]	0 [0]		100	0	0	0
Lung	Lung carcinoma	143 [99]	1 [1]	0 [0]	0.5972 (*n*.*s*.)	99	0	1	0
	Normal lung	40 [100]	0 [0]	0 [0]		100	0	0	0
Stomach	Gastric carcinoma	14 [82]	1 [6]	2 [12]	0.0735 (*n*.*s*.)	82	0	0	18
	Gastric inflammatory lesions	38 [90]	4 [10]	0 [0]		90	7	0	2
Breast	Breast carcinoma	162 [80]	33 [16]	8 [4]	0.8514 (*n*.*s*.)	80	0	0	19
	Normal breast	6 [86]	1 [14]	0 [0]		86	0	0	14

Tissues were scored as unstained (0+), low NOX1 expressers (1+), or high NOX1 expressers (2+, 3+). Percentage of tissues per score is shown in brackets. *n*.*s*., not statistically significant.

^1^The Chi-square statistical test was used to determine significant differences between the proportions of low (1+) and high expressers (2+, 3+) between malignant tissue and matched or adjacent normal tissue.

A representative image from a moderately differentiated adenocarcinoma of the colon displayed non-uniform, strong cytoplasmic NOX1 protein staining in colon cancer epithelial cells (Fig 5A). NOX1 protein staining was strongest toward the luminal surface of the cells in colonic tubular adenomas, whereas it was weak to moderate in hyperplastic polyps along the apical and luminal surface of the cells. A representative section from a small intestinal adenocarcinoma demonstrated moderately strong cytoplasmic NOX1 immunostaining with a more concentrated expression in the luminal surface of the glands (Fig 5B). Moreover, twelve percent of patients with gastric carcinoma had moderate to high NOX1 staining compared to none of the patients with non-malignant gastric inflammatory lesions (*p =* 0.07, Table 3). In gastric adenocarcinoma, NOX1 staining was strong in the apical and luminal surfaces of the malignant cells (Fig 5C). Overall, the staining of NOX1 protein in the matched normal/non-neoplastic tissues from the stomach was either absent or very weakly luminal in the surface epithelium as shown for the normal.

**Fig 6:**
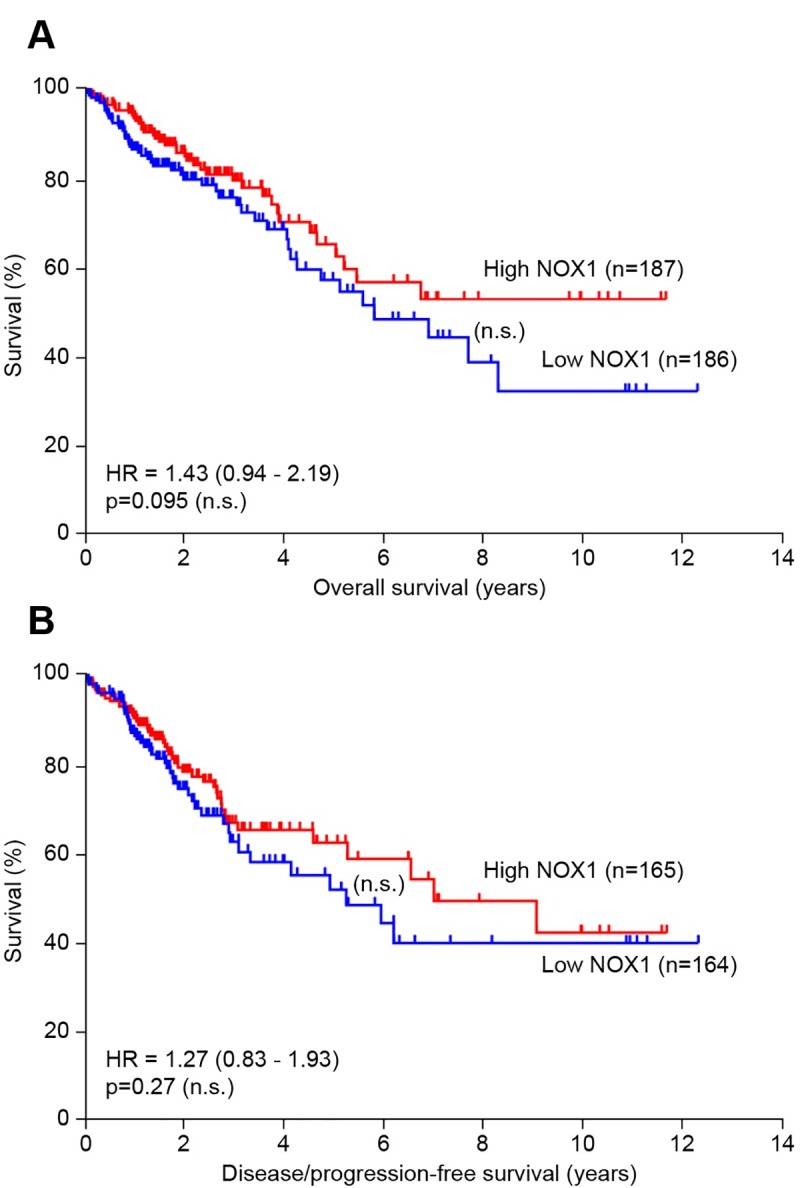
NOX1 expression and patient survival. In the TCGA colorectal adenocarcinoma dataset, 373 patients had known NOX1 mRNA and overall survival information; 329 patients had known NOX1 mRNA and disease/progression-free survival. Kaplan-Meier curves displaying **(A)** overall survival and **(B)** disease/progression-free survival of these patients stratified into high and low NOX1 mRNA expressers. **(A, B)** The median NOX1 expression value was used as a cutoff, and no significant differences in overall survival or progression free survival were detected between high and low NOX1 expressers.

These observations led us to interrogate possible associations between NOX1 expression and patient survival. We performed a Kaplan-Meier analysis and Cox regression of next-generation sequencing data from TCGA colorectal adenocarcinoma cohort. The dataset was stratified into 2 groups representing high and low NOX1 mRNA expressers, with the median NOX1 expression value serving as a cutoff. No significant association was found between NOX1 mRNA expression and either overall survival or progression free survival (Fig 6A and 6B). These results, if confirmed, suggest that NOX1 may contribute to an earlier stage of CRC carcinogenesis rather than to the course of advanced disease.

## Discussion

NOX-associated ROS and oxidative stress have been implicated in the pathogenesis of multiple cancers, including gastrointestinal malignancies [8, 16, 23]. Accumulating evidence suggests that NOXs promote oxidative damage to DNA, contributing to genomic instability, as well as enhance normoxic expression of HIF-1α and VEGF-A, promoting angiogenesis [29, 54]. However, study of the biological functions of NOX proteins has been constrained by the lack of relevant, validated antibodies to confirm the presence of specific NOX isoforms in various human tissues and tumors [55]. The majority of commercially available and academically-produced primary antibodies used for the immunodetection of NOX proteins are polyclonal [56–59]. Although it is more economical to produce polyclonal antibodies, these are associated with several limitations, including high variability between batches and potential for cross-reactivity.

To overcome these limitations and to facilitate the study of NOXs in human tumors, we have recently developed and carefully validated monoclonal antibodies highly specific for human DUOX, NOX5, and NOX4, which allowed us to demonstrate the prevalence of these NOX isoforms at the protein level across a wide range of human malignancies [35, 36, 60]. We have since made these antibodies available for research use by other laboratories in the field. In the current effort, a NOX1 mouse monoclonal antibody was raised against a truncated, codon-optimized, recombinant protein comprising of 341 amino acids (residues 224–564) at the C-terminus intracellular portion of human NOX1 (S1 Fig, S2 Fig). We demonstrated herein that our NOX1 monoclonal antibody was specific, selective, and could readily be used for Western blot analysis, flow cytometry, confocal microscopy, and immunohistochemical detection of the NOX1 protein with high specificity in both constitutive and recombinant NOX1 expression settings (Fig 1B, 1D, 1E and 1F; Fig 2B; Fig 5; S3 and S4 Figs).

Using this novel immunological tool, we have identified specific cellular systems suitable for the study of NOX1 tumor biology. We confirmed that in the genetically-engineered stable overexpression system HEK293-NOX1, the membrane localization of NOX1 protein mirrored that of endogenous NOX1 expression (S3 Fig). Furthermore, we found that NOX1 protein expression detected with our mAb is highly correlated with ROS production in specific cell lines (Fig 2B and Fig 3B). The recombinant NOX1 protein expressed in HEK293-NOX1 cells, as well as the NOX1 protein in the LS513 line, was functional, with enzymatic activity commensurate with NOX1 mRNA and protein expression (Fig 2). On the other hand, functional testing of NCI-H508 cells that lack the NADPH binding site (Fig 3A) with the luminescence assay revealed a lack of oxidase activity in these cells, consistent with the presence of the non-functional splice variant *NOX1-S*/*NOX1-Lv* [38, 61]. Hence, it is imperative that functional NOX1 activity be demonstrated, in addition to expression levels, before studies of NOX1 biology at the cellular level are initiated. We also provided an extensive characterization of NOX1 expression and functionality in commercially available CRC cell lines, such as the LS513 cell line which has the highest NOX1 expression level after the HEK293-NOX1 overexpression system (Fig 2B and Fig 3B).

LS513 cells are TP53 wild type and KRAS G12D mutant. Prior studies have linked oncogenic RAS mutations with NOX1 overexpression [46, 62]. RAS is a cancer driver gene that, when mutated, promotes uncontrolled cell growth and increased cell survival [63]. A third of human cancers harbor an oncogenic RAS mutation; pancreatic, colorectal, and lung cancers demonstrate the highest prevalence of mutated RAS [63–65]. Over a decade ago, Mitsushita *et al*. provided evidence directly implicating the flavoprotein NOX1 as the source of oncogenic RAS-dependent ROS [46]. The authors suggested that oncogenic RAS upregulated the expression of NOX1 in normal rat kidney (NRK) epithelial cells and in NIH 3T3 mouse fibroblasts via the mitogen-activated protein kinase (MAPK) pathway, and that cellular transformation could be prevented by the knockout of NOX1 with a specific siRNA. In their studies, the flavoprotein inhibitor diphenylene iodonium (DPI) and the sulfhydryl donor *N*-acetylcysteine (NAC) readily suppressed mitogenic signaling, establishing a relationship between NOX1-derived ROS and malignant transformation by oncogenic RAS [46, 66].

However, that report using non-human model cell lines contrasts with our finding that RAS mutation status is not significantly correlated with NOX1 mRNA expression in human transformed/cancer cells of the ATCC repository and the CCLE database (Fig 4). On the other hand, we have demonstrated significant overexpression of NOX1 mRNA in tumor specimens with both KRAS wild type (1.6-fold increase, *p<0.05), and KRAS mutant (1.9-fold increase, ***p<0.001) genotypes when these CRCs are compared to specimens of normal colonic mucosa derived from the TCGA cohort (Fig 4). These results are in agreement with the report by Laurent *et al*. of NOX1 mRNA being upregulated in 57% of a 24 patient colon cancer cohort that was compared to adjacent normal colonic tissue from the same patients, a trend that correlated with activating mutations in KRAS codons 12 and 13 [62]. We did not find a relationship between the levels of NOX1 and KRAS expression at the mRNA level, however. Taken together, these data suggest that oncogenic KRAS and NOX1 may together contribute to malignant colonic transformation *in vivo*, although the direct mechanism(s) for such an interaction remains to be determined in patient materials.

Although cell-based studies are informative, as suggested by the conflicting association of mutant RAS with CRC cell lines versus primary tumors just outlined, these models have limitations and may not fully recapitulate the biology of human tumors [67–69]. In part, this is due to adaptation to two-dimensional culture conditions through epigenetic and transcriptional reprogramming [70]. Therefore, it is important to confirm *in vitro* observations in relevant human cancer specimens. In this study, we systematically assessed the prevalence of NOX1 in multiple human cancers using appropriate samples sizes to draw statistically robust conclusions. We have evaluated over 1,200 human tumors and inflammatory diseases (including 146 colon cancers and 44 adenomatous polyps; and large sample sizes of small intestinal, prostate, ovary lung, stomach, and breast carcinomas) to define the prevalence of NOX1 expression in these diseases at a pre-defined level of statistical certainty. We clearly demonstrate high levels of NOX1 protein expression in more than a third of colon cancers and colonic polyps compared to normal colonic tissues. We also demonstrate high level NOX1 expression for the first time in adenocarcinomas of the small intestine (Fig 5, Table 3). When detected, NOX1 expression was primarily localized to the cytoplasm of epithelial cells towards the luminal surface, consistent with the design of our antibody, which was made to recognize an intracellular epitope on the NOX1 protein (S1A Fig**)**. Furthermore, although some studies have suggested a role for NOX1 in gastric, breast, and prostate cancers [71–73], our immunohistochemical evaluation of NOX1 protein revealed that NOX1 was not overexpressed in a large percentage of the tumor specimens we examined with these diagnoses. Twelve percent of gastric carcinomas, 4% of breast carcinomas, and 2% of prostate carcinomas were scored as high NOX1 expressers (2+ and 3+; Table 3). These results suggest that NOX1 may play a more limited role in the pathogenesis of these diseases.

In summary, we developed a validated, sensitive, and isoform-specific mouse monoclonal antibody for the study of NOX1 in human malignancies (available for research use upon request). Although we did not find a significant correlation between NOX1 expression and oncogenic KRAS mutations in CRC cell lines, RAS mutations do appear to be correlated with a significant increase in NOX1 mRNA expression in primary colon cancer surgical specimens (using data obtained from TCGA). In the present work, we have provided additional evidence that NOX1 is a relevant molecular target in gastrointestinal malignancies by demonstrating overexpression in clinical colorectal and small intestinal tumors at the protein level. We predict that the immunologic tool described in this study will be used to help dissect the mechanisms regulating NOX1 expression and resulting tumorigenic molecular pathways, as well as to further evaluate the role of NOX1 expression in patients with pre-malignant adenomatous polyps in the colon.

## Supporting information

S1 Fig NOX1 monoclonal antibody development. **(A)** Schematic representation of the conserved structural features of the NOX1 protein: 6 transmembrane domains, and cytosolic FAD and NADPH binding domains. The antibody epitope spans the FAD and NADPH binding domains, and the structural model (cyan) was built by the SWISS-MODEL server using the experimental crystal structure for the dehydrogenase domain of *Cylindrospermum stagnale* NADPH oxidase 5 (NOX5) as a template (PDB code: 5o0x:A). The aligned portions of human NOX1 and the template share 43% sequence identity. **(B)** Alignment of the human NOX1 amino acid region expressed for antigen development (AA 224–564) with the mouse ortholog. Regions of highest antigenicity were predicted by EMBOSS Antigenic and are highlighted in red font.
(TIF)

S2 Fig Sequence alignment of the NADPH- and flavin- binding region of human NOX proteins. **(A)** The amino acids comprised in the NADPH- and flavin-binding region of human NOX proteins were aligned using Clustal Omega. For NOX1, this region covers residues 224 564 in NOX1. Asterisk (*), residue is fully conserved across the 7 NOX sequences; colon (:), conservation between amino acids with strongly similar physicochemical properties; period (.), conservation between amino acids with weakly similar properties; blank (), position is not conserved across the 7 NOX proteins. **(B)** Number and proportion of identical amino acids between the NADPH- and FAD-binding region of NOX1 and other human NOXs.
(TIF)

S3 Fig Membrane localization of NOX1 protein in LS513 and HEK293-NOX1 cell lines. **(A, B)** NOX1 was detected in the membrane fraction of **(A)** HEK293-NOX1 clones, and in **(B)** parental LS513 cells and LS513 cells transfected with NOX1-siRNA. HSP90, Na/K ATPase, lamin A/C, and vimentin were used as markers of subcellular compartments. F1: cytosol; F2: membrane; F3: nucleus; F4: cytoskeleton.
(TIF)

S4 Fig Flow cytometric detection of NOX1 in HEK293-vector and HEK293-NOX1 clones. **(A)** HEK293 cells stably transfected with either a vector control (HEK293-vector) or the pCMV-NOX1 plasmid (HEK293-NOX1) were fixed, permeabilized, and labeled with 2 μg/ml purified NOX1 mAb. The cells stained with the NOX1 antibody were labeled with AF-488 goat anti-mouse antibody (1:1000), and the fluorescence was detected by flow cytometry. Representative figures from at least 3 experiments are displayed. Unstained cells (red) and cells stained with irrelevant mouse IgG mAb (turquoise and light green) represent background staining controls. **(B)** Flow cytometric detection of NOX1 in non-permeabilized cells.
(TIF)

S5 Fig Detection of *NOX1-L* and *NOX1-S*/*NOX1-Lv* in transfected HEK293 clones. HEK293 cells were transfected with either the pCMV-*NOX1-L* plasmid (full length NOX1), pCMV-*NOX1-Lv* plasmid (variant/short form NOX1), or an empty vector. Transiently transfected (#) cells were collected after 48 h of transfection, while stable pooled (§) clones for *NOX-L* and *NOX1-Lv* transfected cells were obtained subsequent to selection with puromycin. NOX1 expression was confirmed **(A)** at the mRNA level by RT-PCR in both transient (#) and stable pooled (§) clones of HEK293-transfected *NOX1-L* and *NOX1-Lv* cells (***p<0.001 vs. untransfected cells). NOX1 mRNA level is given relative to β-actin. **(B)** Western blot analysis confirmed the detection of full length *NOX1-L* by the NOX1 antibody (lanes 3 and 4), with no/minimal detection of NOX1 in either the transient (#) or stable (§) *NOX1-Lv* generated HEK293 cells (lanes 5 and 6), despite NOX1 mRNA levels being comparable in both *NOX1-L* and *NOX1-Lv* transfected cells (see S5A Fig). The expression of NOX1 in LS513 cells was used as a positive control. **(C)** Absence of *NOX1-Lv* immunodetection in HEK293 stable pooled (§) clones. HEK293-*NOX1-Lv* and HEK293-vector control cells were evaluated for detection of NOX1 by confocal microscopy under conditions similar to those of Fig 1E. The cells were immunostained with NOX1 mouse mAb (green). Cell nuclei were stained with 4′,6-diamidino-2-phenylindole (DAPI; blue). Digital images were taken at 63X magnification.
(TIF)

S1 Table ELISA screening and isotyping of 3 positive hybridoma clones using HNC immunogen and His-tag. (PDF)

S2 Table Spearman and pearson correlation between the expression of NOX1 and KRAS in colon cancer cell lines of the ATCC and CCLE, and in human colorectal tumor specimens from TCGA. (PDF)

S1 File Graphical abstract. (TIF)
