# Supplementary figures and images for: NADPH oxidase 1 is highly expressed in human large and small bowel cancers
Source: PLoS One. 2020 May 19;15(5):e0233208. doi: 10.1371/journal.pone.0233208 (PMC7237001; doi:10.1371/journal.pone.0233208)

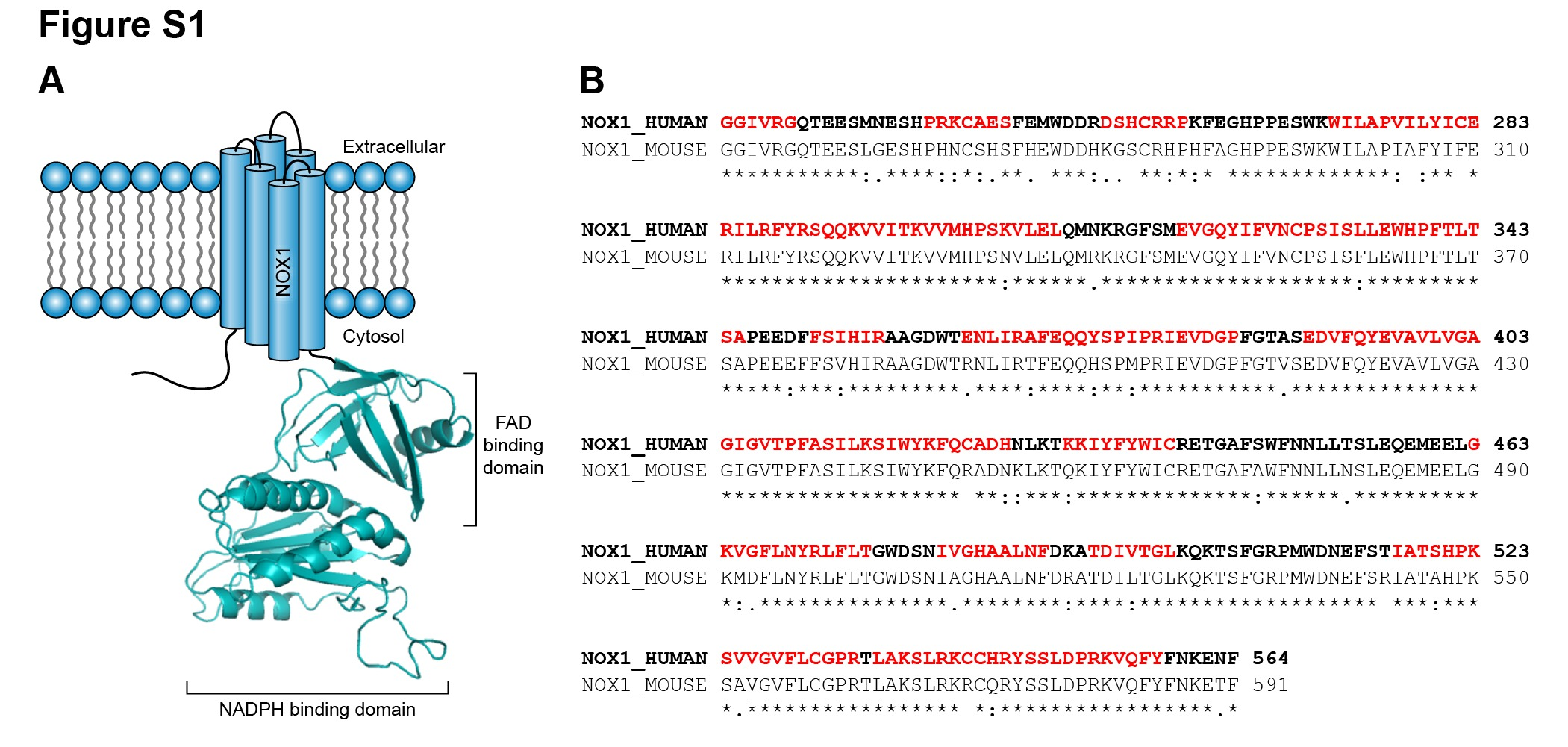

Supplement: S1 Fig — (A) Schematic representation of the conserved structural features of the NOX1 protein: 6 transmembrane domains, and cytosolic FAD and NADPH binding domains. The antibody epitope spans the FAD and NADPH binding domains, and the structural model (cyan) was built by the SWISS-MODEL server using the experimental crystal structure for the dehydrogenase domain of Cylindrospermum stagnale NADPH oxidase 5 (NOX5) as a template (PDB code: 5o0x:A). The aligned portions of human NOX1 and the template share 43% sequence identity. (B) Alignment of the human NOX1 amino acid region expressed for antigen development (AA 224–564) with the mouse ortholog. Regions of highest antigenicity were predicted by EMBOSS Antigenic and are highlighted in red font. (TIF) [file pone.0233208.s001.tif]

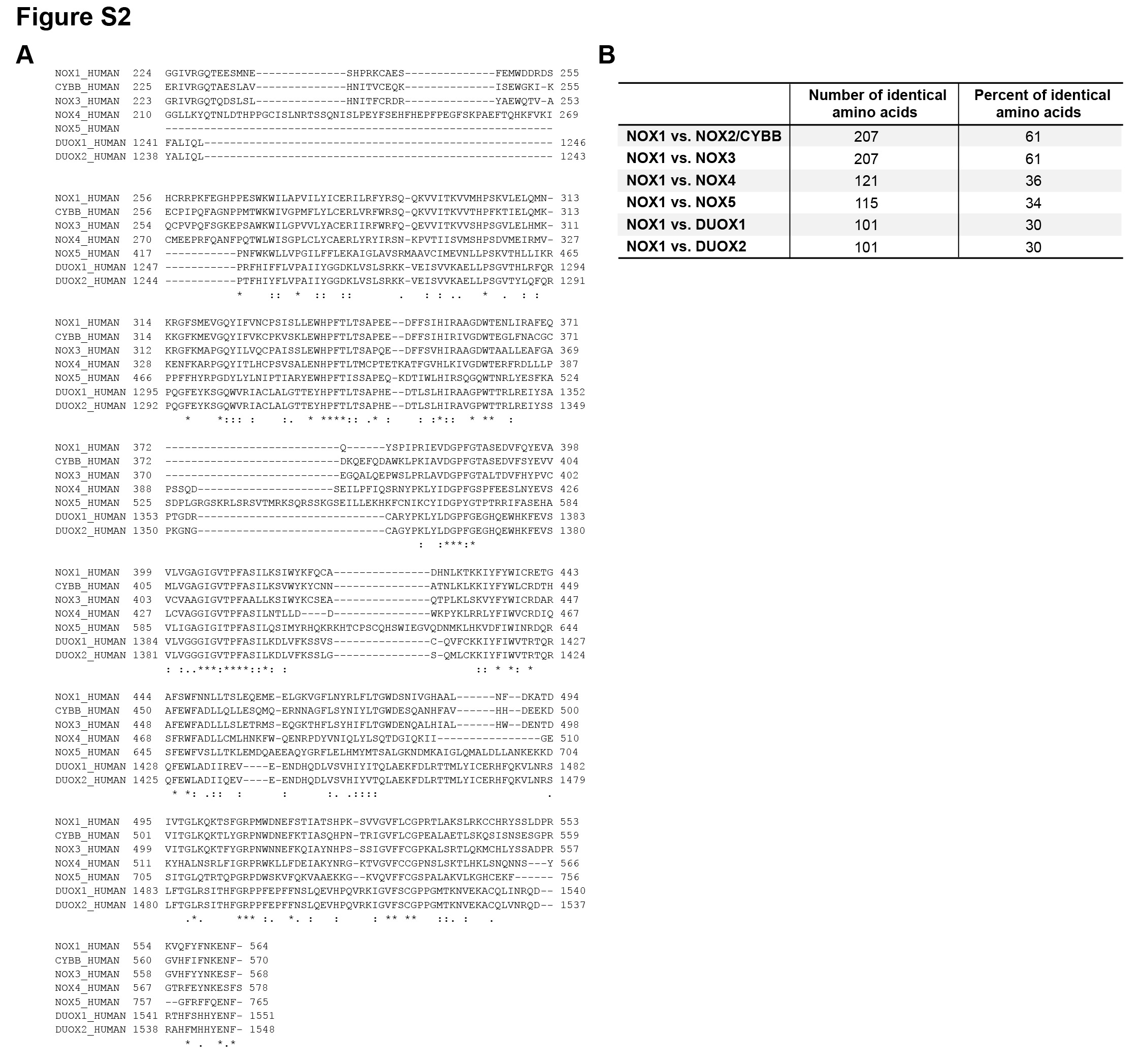

Supplement: S2 Fig — (A) The amino acids comprised in the NADPH- and flavin-binding region of human NOX proteins were aligned using Clustal Omega. For NOX1, this region covers residues 224 564 in NOX1. Asterisk (*), residue is fully conserved across the 7 NOX sequences; colon (:), conservation between amino acids with strongly similar physicochemical properties; period (.), conservation between amino acids with weakly similar properties; blank (), position is not conserved across the 7 NOX proteins. (B) Number and proportion of identical amino acids between the NADPH- and FAD-binding region of NOX1 and other human NOXs. (TIF) [file pone.0233208.s002.tif]

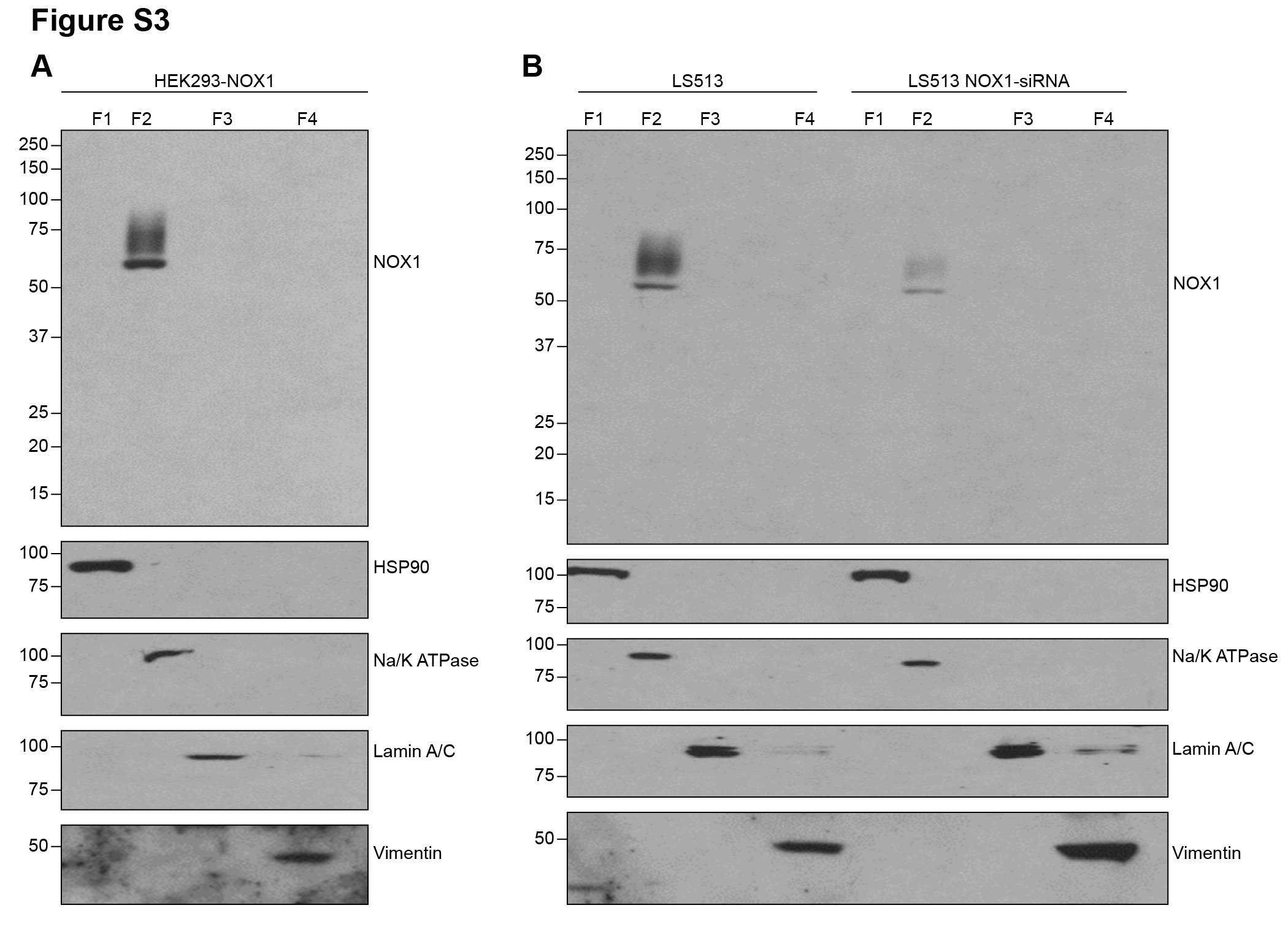

Supplement: S3 Fig — (A, B) NOX1 was detected in the membrane fraction of (A) HEK293-NOX1 clones, and in (B) parental LS513 cells and LS513 cells transfected with NOX1-siRNA. HSP90, Na/K ATPase, lamin A/C, and vimentin were used as markers of subcellular compartments. F1: cytosol; F2: membrane; F3: nucleus; F4: cytoskeleton. (TIF) [file pone.0233208.s003.tif]

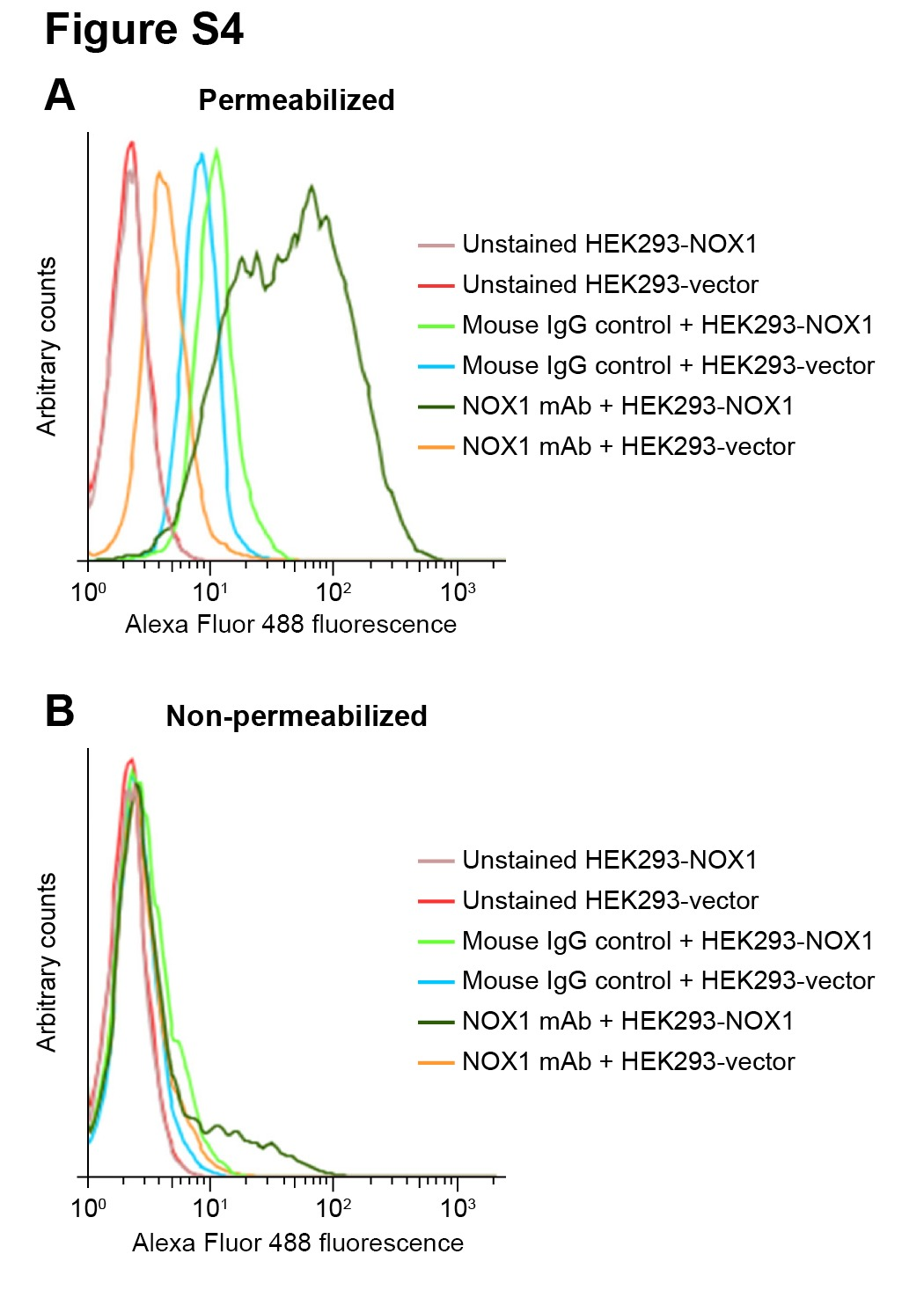

Supplement: S4 Fig — (A) HEK293 cells stably transfected with either a vector control (HEK293-vector) or the pCMV-NOX1 plasmid (HEK293-NOX1) were fixed, permeabilized, and labeled with 2 μg/ml purified NOX1 mAb. The cells stained with the NOX1 antibody were labeled with AF-488 goat anti-mouse antibody (1:1000), and the fluorescence was detected by flow cytometry. Representative figures from at least 3 experiments are displayed. Unstained cells (red) and cells stained with irrelevant mouse IgG mAb (turquoise and light green) represent background staining controls. (B) Flow cytometric detection of NOX1 in non-permeabilized cells. (TIF) [file pone.0233208.s004.tif]

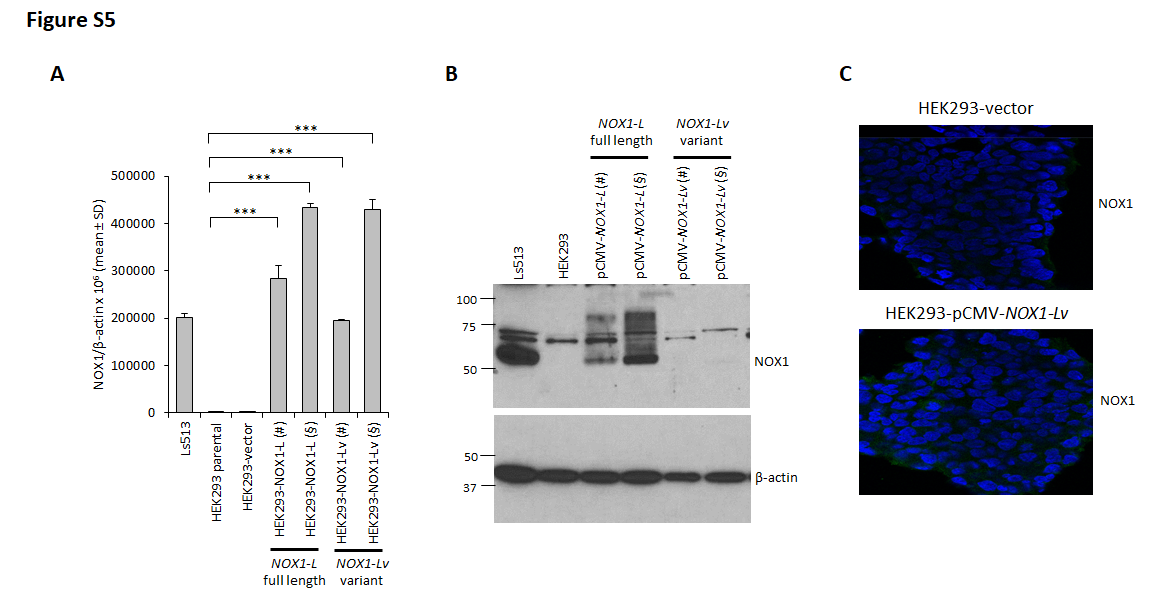

Supplement: S5 Fig — HEK293 cells were transfected with either the pCMV-NOX1-L plasmid (full length NOX1), pCMV-NOX1-Lv plasmid (variant/short form NOX1), or an empty vector. Transiently transfected (#) cells were collected after 48 h of transfection, while stable pooled (§) clones for NOX-L and NOX1-Lv transfected cells were obtained subsequent to selection with puromycin. NOX1 expression was confirmed (A) at the mRNA level by RT-PCR in both transient (#) and stable pooled (§) clones of HEK293-transfected NOX1-L and NOX1-Lv cells (***p<0.001 vs. untransfected cells). NOX1 mRNA level is given relative to β-actin. (B) Western blot analysis confirmed the detection of full length NOX1-L by the NOX1 antibody (lanes 3 and 4), with no/minimal detection of NOX1 in either the transient (#) or stable (§) NOX1-Lv generated HEK293 cells (lanes 5 and 6), despite NOX1 mRNA levels being comparable in both NOX1-L and NOX1-Lv transfected cells (see S5A Fig). The expression of NOX1 in LS513 cells was used as a positive control. (C) Absence of NOX1-Lv immunodetection in HEK293 stable pooled (§) clones. HEK293-NOX1-Lv and HEK293-vector control cells were evaluated for detection of NOX1 by confocal microscopy under conditions similar to those of Fig 1E. The cells were immunostained with NOX1 mouse mAb (green). Cell nuclei were stained with 4′,6-diamidino-2-phenylindole (DAPI; blue). Digital images were taken at 63X magnification. (TIF) [file pone.0233208.s005.tif]

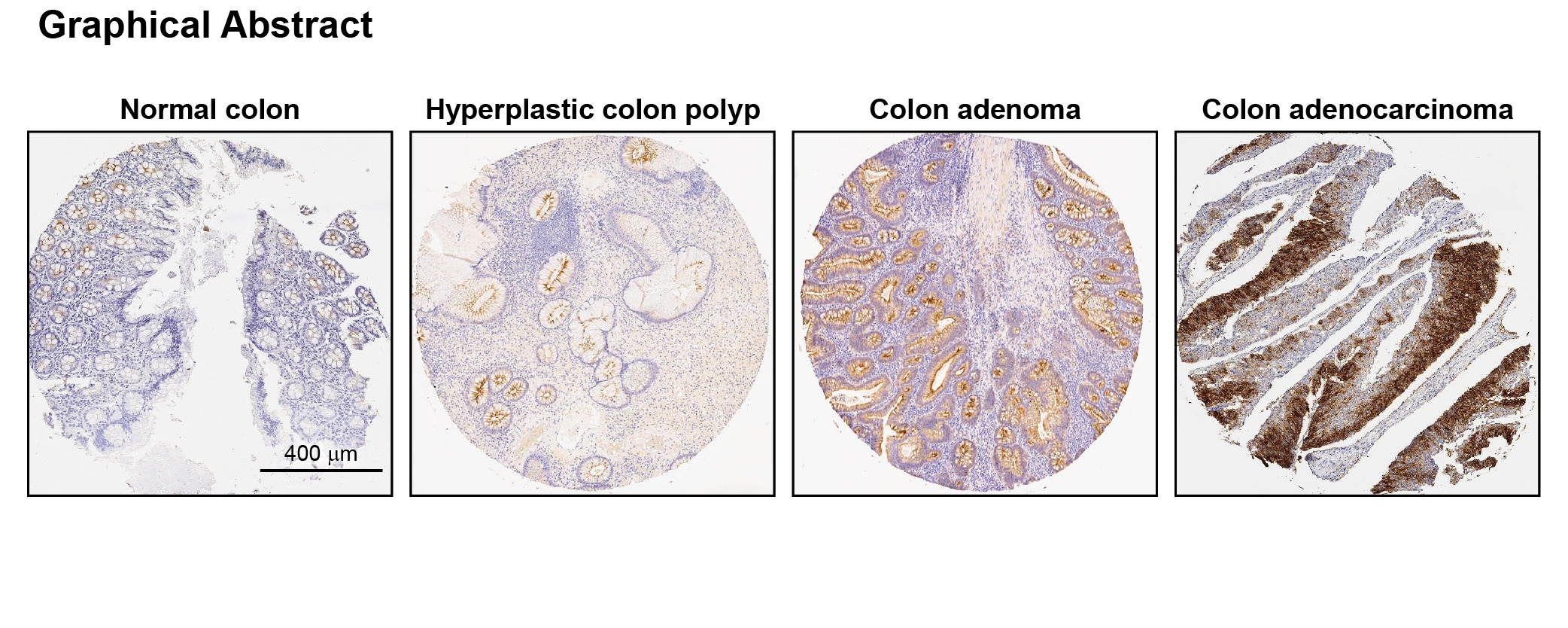

Supplement: S1 File — (TIF) [file pone.0233208.s008.tif]
