# Supplementary material for: NADPH oxidase 1 is highly expressed in human large and small bowel cancers
Source: PLoS One. 2020 May 19;15(5):e0233208. doi: 10.1371/journal.pone.0233208 (PMC7237001; doi:10.1371/journal.pone.0233208)
Supplement: S1 Table — (PDF) [file pone.0233208.s006.pdf]

S1 Table. ELISA screening and isotyping of 3 positive hybridoma clones using HNC immunogen and His-tag.

Clone No.	HNC-His	His-tag	Isotype
#4	1.197	0.111	G1
#22	1.227	0.082	G2b
#55	1.223	0.027	G2a
Blank control	0.081	0.09	-
Negative control	0.037	0.047	-
Positive control	-0.048	1.025	-
