# Supplementary material for: NADPH oxidase 1 is highly expressed in human large and small bowel cancers
Source: PLoS One. 2020 May 19;15(5):e0233208. doi: 10.1371/journal.pone.0233208 (PMC7237001; doi:10.1371/journal.pone.0233208)
Supplement: S2 Table — (PDF) [file pone.0233208.s007.pdf]

S2 Table. Spearman and Pearson correlation between the expression of NOX1 and KRAS in colon cancer cell lines of the ATCC and CCLE, and in human colorectal tumor specimens from TCGA. NOX1 and KRAS gene expression are not significantly correlated in the 3 databases.

	**Spearman correlation**		**Pearson correlation**	
	**rho**	***p* value**	**r**	***p* value**
ATCC (n=27)	0.14	0.4846	-0.09	0.6481
CCLE (n=62)	0.06	0.6459	0.13	0.3280
TCGA (n=623)	0.06	0.1701	0.04	0.3485
